# Protein phosphatase 1 regulatory subunit 15 A promotes translation initiation and induces G2M phase arrest during cuproptosis in cancers

**DOI:** 10.1038/s41419-024-06489-w

**Published:** 2024-02-16

**Authors:** Chunyu Liu, Liang Chen, Yukun Cong, Lulin Cheng, Yujun Shuai, Fang Lv, Kang Chen, Yarong Song, Yifei Xing

**Affiliations:** Department of Urology, Union Hospital, Tongji Medical College, Huazhong University of Science and Technology, 430022 Wuhan, China

**Keywords:** Cancer, Cell death

## Abstract

Copper ions play a crucial role as cofactors for essential enzymes in cellular processes. However, when the intracellular concentration of copper ions exceeds the homeostatic threshold, they become toxic to cells. In our study, we demonstrated that elesclomol, as a carrier of copper ions, caused an upregulation of protein phosphatase 1 regulatory subunit 15 A (PPP1R15A), which plays a role in regulating substrate selectivity of protein phosphatase 1 during cuproptosis. Mechanistically, we investigated that PPP1R15A activated translation initiation by dephosphorylating eukaryotic translation initiation factor 2 subunit alpha at the S51 residue through protein phosphatase 1 and phosphorylating eukaryotic translation initiation factor 4E binding protein 1 at the T70 residue. In addition, PPP1R15A reduced H3K4 methylation by altering the phosphorylation of histone methyltransferases, which led to the silencing of MYC and G2M phase arrest.

## Introduction

Copper ions play a crucial role as cofactors for enzymes involved in various cellular physiological functions, such as mitochondrial respiration, signal transduction, antioxidant defense, and biosynthesis of small molecules [1–3]. However, when the intracellular concentration of copper ions exceeds the homeostatic threshold, it can lead to cell death, known as cuproptosis [4]. Copper-based treatment has presented new opportunities for treatment of chemotherapy-insensitive tumors [5, 6]. Previous clinical trials have demonstrated the safety and selective cytotoxicity of elesclomol, an effective copper ion carrier, towards tumor cells [7]. The anticancer effect of elesclomol relies on its ability to transport copper ions and the intensity of mitochondrial metabolism in cancer cells [8, 9]. According to a melanoma trial, elesclomol generates excessive reactive oxygen species in cells and causes cell death [10]. Another study finds that elesclomol-induced cell death involves DNA damage and cell cycle arrest [11]. Recently, Golub et al. demonstrates that elesclomol, by carrying copper into cells, induces mitochondrial dysfunction and cuproptosis through ferredoxin 1 (FDX1) [12]. They confirm that excessive intracellular copper causes mitochondrial damage that results in proteotoxic stress and ultimately cell death, and propose that the cell death mechanism be termed cuproptosis. However, the specific mechanism of how mitochondrial damage causes proteotoxic stress remains unclear.

Rapid protein anabolism not only meets the needs of vigorous proliferation, but also brings the vulnerability of protein homeostasis in tumor cells. It has been proved that the heat shock protein 70 (HSP70) as a marker of proteotoxic stress significantly increases in cuproptosis by Golub et al. HSP70 is involved in the co-translational folding of nascent proteins, and its expression level can reflect the rate of nascent protein synthesis [13]. It is well-known that up to 30% of new proteins or peptides are misfolded and newly synthesized polypeptides are especially prone to aggregation [14, 15]. Furthermore, mitochondria under stress is proven to constantly interact with the nucleus and cytoplasm to activate transcription, translation and post-translational processes, aiming to restore normal mitochondrial function [16]. We hypothesized that cells activated translation initiation under mitochondrial damage, leading to the accumulation of unfolded and misfolded proteins, ultimately resulting in cuproptosis. Translation initiation in eukaryotes is regulated by key regulators such as eukaryotic translation initiation factor 2 subunit alpha (EIF2S1) and eukaryotic translation initiation factor 4E (EIF4E). Phosphorylation of EIF2S1 suppresses global translation, while phosphorylation of eukaryotic translation initiation factor 4E binding protein 1 (4EBP1) releases EIF4E and initiates translation [17].

Protein phosphorylation is one of the most common post-translational modifications that influences a significant portion of cellular proteins and processes [18, 19]. The mammalian genome encodes fewer than 40 protein Ser/Thr phosphatases that offset several hundred Ser/Thr kinases [20]. In eukaryotes, protein Ser/Thr phosphatases, particularly protein phosphatases 1 (PP1) and 2 A (PP2A), play a major role in dephosphorylation [21]. The phosphatases have numerous regulatory subunits that form stable protein-protein complexes, affecting substrate selectivity and performing diverse functions [22]. Protein Phosphatase 1 Regulatory Subunit 15 A (PPP1R15A), a stress-induced protein, promotes dephosphorylation of EIF2S1, TGF beta type I receptor and I-κ B kinase through PP1 [23–25]. PPP1R15A has been implicated in various cell death signals and can initiate or enhance cell death [26]. In the past, researchers have focused on the dephosphorylation caused by PPP1R15A, but they have neglected that PPP1R15A can regulate the substrate selection of PP1 to maintain some proteins phosphorylation.

Epigenetic regulation can silence gene expression at specific genomic regions through chromatin remodeling either by modifying higher order chromatin fiber structure, nucleosomal histones, or the cytosine DNA methylation. Histone H3 lysine 4 methylation (H3K4me) is generally considered to be a gene activation signal, which plays an important role in transcription initiation and elongation. It has been confirmed that the level of H3K4me is positively correlated with MYC expression in myeloid malignancies and lymphomas [27, 28]. Reduction of H3K4me has been shown to induce G2M phase arrest in various cancers including breast cancer, gastric cancer and prostate cancer, etc [29–32]. Lysine methyltransferase, such as KMT2A, KMT2B and KMT2D, are important regulators of H3K4me [33]. Phosphorylation status can affect the activity of these methyltransferases. KMT2A is phosphorylated at serine 516 by ATR serine/threonine kinase which disrupts its interaction with E3 ligase leading to its accumulation [34]. KMT2B is phosphorylated by cyclin dependent kinase 2 to facilitate its recruitment to developmental genes [35]. AKT binds and phosphorylates KMT2D, attenuating methyltransferase activity in ER-positive breast cancer [36].

In our study, we discovered that elesclomol facilitates the transport of copper ions into cells, leading to mitochondrial stress and activation of PPP1R15A. Through the utilization of phosphoproteomics and transcriptomics, we were able to validate that PPP1R15A enhances translation initiation, thereby promoting proteotoxic stress by regulating the phosphorylation of EIF2S1 and 4EBP1. Additionally, we observed that PPP1R15A reduces H3K4me levels, resulting in the suppression of *MYC* and its associated genes by modulating the phosphorylation of KMT2A/B/D, which subsequently induces G2M phase arrest.

## Materials and methods

### Reagents

Elesclomol (STA-4783), Puromycin (CL13900), Ferrostain-1 (S7243), and Tetrathiomolybdate (E1166) were purchased from Selleck Chemicals (China). Z-VAD-FMK (HY-16658B), N-Acetylcysteine (HY-B0215) and Necrostatin-1 (HY-15760) were purchased from MedChemExpress (China).

### Cell culture

Human cancer cell lines PC-3, DU145, HeLa, A549, BT-549, and HEK293T were purchased from the Cell Bank of the Chinese Academy of Sciences (Shanghai, China). PC-3, DU145, HeLa, and BT-549 were cultured in Roswell Park Memorial Institute (RPMI) 1640 medium (Hyclone, USA) supplemented with 10% Fetal bovine serum (FBS) (Biological Industries, Israel). A549 and HEK293T were cultured in Dulbecco’s Modified Eagle Medium (DMEM) (Hyclone) supplemented with 10% FBS. In this study, all cells were maintained at 37 °C in a 5% CO2 incubator. All cell lines were confirmed within 6 months before use by using a short tandem repeat profiling and were confirmed negative for Mycoplasma contamination.

### Viability and cell proliferation assays

The cell Counting Kit-8 (CCK-8) assay (Dojindo Laboratories, Japan) and BeyoClick™ EdU Cell Proliferation Kit (Beyotime, China) were used to determine cell viability after elesclomolpulse treatment. The results were obtained according to the manufacturer’s instructions. For elesclomol treatment, media were supplemented with 1 μM CuCl_2_, unless otherwise specified, and pulse treatment was incubated for 2 hours.

### Quantitative real‑time PCR (qRT‑PCR)

Total RNA was extracted using the TRIzol reagent (Invitrogen, USA) and reverse-transcribed into cDNA using the PrimeScript RT Reagent Kit (Takara, Japan). To quantify expression levels, qRT-PCR was performed on a StepOnePlus™ Real-Time PCR System (Applied Biosystems, USA). The 2–ΔΔCT method was used to calculate the relative levels of mRNA. Actin Beta (ACTB) was used as an internal control. Primers used in this study were synthesized by Sangon Biotech. Primers used for mRNA expression were:

HSPA9: 5’-CTTGTTTCAAGGCGGGATTATGC-3’; 5’-GCAGGAGTTGGTAGTACCCAAA-3’

MYC: 5’-GCTGCTTAGACGCTGGATTT-3’; 5’-TAACGTTGAGGGGCATCG-3’

FOXM1: 5’-CGTCGGCCACTGATTCTCAAA-3’; 5’-GGCAGGGGATCTCTTAGGTTC-3’

Cyclin B: 5’-AATAAGGCGAAGATCAACATGGC-3’; 5’-TTTGTTACCAATGTCCCCAAGAG-3’

CDKN2B: 5’-TCAGTCGGCTTCCGAGGTA-3’; 5’-GTTGCGTGGTTCAATGCC-3’

P21: 5’-TGTCCGTCAGAACCCATGC-3’; 5’-AAAGTCGAAGTTCCATCGCTC-3’

ACTB: 5’-TGGCACCCAGCACAATGAA-3’; 5’-CTAAGTCATAGTCCGCCTAGAAGCA-3’

### Western blotting

Total protein was extracted from cells using Radioimmunoprecipitation assay (RIPA) lysis buffer (Servicebio, China) and protein concentration was tested using a BCA Protein Assay Kit (Beyotime). SDS-PAGE sample-loading buffer (ThermoFisher Scientific, USA) was added, and samples were stored at −20 °C. Subsequently, electrophoresis was completed in 10-12% SDS-PAGE gels and then transferred to a Polyvinylidene fluoride (PVDF) membrane (Millipore). Next, the membranes were blocked in 5% skim milk or bovine serum albumin (BSA) for 1–2 hours at RT. Membranes were washed with tris-buffered saline (TBST) three times for 10 min each and membranes were incubated with their corresponding antibodies overnight at 4 °C. This was followed by hybridization with a specific horseradish peroxidase (HRP)-conjugated secondary antibody for 1 hour at RT. Finally, the membranes were visualized using an Enhanced Chemiluminescence (ECL) Substrate Kit (Millipore), and images were obtained using a Bio Spectrum 600 Imaging System (UVP). The following antibodies were used: HRP conjugated Affinipure Goat anti-mouse antibody, (SA00001-1, Proteintech), HRP conjugated Affinipure Goat anti-rabbit antibody, (SA00001-2, Proteintech), Anti-Puromycin (EQ0001, Kerafast) Anti-GAPDH (60004-1-Ig, Proteintech), Anti-PPP1R15A (10449-1-AP, Proteintech), Anti-ACO-2 (11134-1-AP, Proteintech), Anti-HSP70 (10995-1-AP, Proteintech), Anti-ACTB (20536-1-AP, Proteintech), Anti-FDX1 (12592-1-AP, Proteintech), Anti-DLAT (4A4-B6-C10, Cell Signaling Technology), Anti-PPP1CC (11082-1-AP, Proteintech), Anti-4EBP1 (60246-1-Ig, Proteintech), Anti-eIF4EBP1 (phospho T70) (ab75831, abcam), Anti-EIF2S1/EIF2A (11170-1-AP, Proteintech), Anti-Phospho-EIF2S1 (Ser51) (28740-1-AP, Proteintech), Anti-H3 (17168-1-AP, Proteintech), Anti-Histone H3 (mono methyl K4) (ab176877, abcam), Anti-Histone H3 (di methyl K4) (ab7766, abcam), Anti-Histone H3 (tri methyl K4) (ab213224, abcam), Anti-SLC31A1 (67221-1-Ig, Proteintech), and Anti-c-MYC (10828-1-AP, Proteintech).

### Copper colorimetric assay

A Copper Colorimetric Assay Kit (E-BC-K300-M, Elabscience, China) and Cell Copper Colorimetric Assay Kit (E-BC-K775-M, Elabscience) were used to detect the copper content of the medium and cells, respectively.

### Flow cytometry

PC-3, DU145, and A549 cells treated under specific conditions (shown in figure legends) were harvested and stained with FITC Annexin V and propidium iodide (PI) (BD Pharmingen). Cells were incubated for 30 min and cell apoptosis was analyzed using flow cytometry (Beckman Coulter, Indianapolis, IN, USA). FlowJo software was used to analyze the results.

PC-3 cells with indicated treatment (shown in figure legends) were collected *via* centrifugation. Precooled phosphate-buffered saline (PBS) was used to resuspend the PC-3 cells and after this they were washed. The cells were then fixed with 70% ethanol overnight. On the second day after centrifugation, precooled PBS was used to resuspend the cells and centrifugation was performed once again. PI was added to the cell precipitate, which was then incubated in the dark for 30 min. The results were analyzed using Modfit software.

### Protein synthesis measurements

To determine protein synthesis in cultured cells, puromycin was added to a medium of 0.5 μg/mL for 30 min before collecting cells. After collecting the cells, follow the normal immunoblotting steps.

### Transmission electron microscopy

Cells were collected after elesclomol treatment and fixed with 2.5% glutaraldehyde at 4 °C. Transmission electron microscopy was performed by Baiqiandu Biotechnology Co., Ltd. (Wuhan, China).

### Plasmid transfection

Three plasmids were purchased from GeneChem (Shanghai, China) for PPP1R15A overexpression, PPP1R15A knockdown, and FDX1 knockdown. The following plasmids were constructed by Vigene Biosciences (Shandong, China): EIF2S1-S51A mutant, EIF2S1-S51D mutant, 4EBP1-T70A mutant, 4EBP1-T70D mutant.

Plasmids and vectors were transfected into cells using Lipofectamine (Invitrogen) according to the manufacturer’s instructions.

### Immunofluorescence staining

Cells were inoculated in a confocal dish and fixed after drug treatment. For DLAT immunofluorescence experiments, the cells were incubated with 100 nM Mitotracker Red CMXRos (M7512, Thermo Fisher Scientific) for 30 min prior to fixation. Cells were permeabilized with 0.3% TritonX-100 for 5 min, blocked with 3% BSA for 30 min at RT, and incubated with primary antibodies overnight at 4 °C. On the second day, cells were incubated with fluorescent-labeled secondary antibodies for 1 hour. Nuclei were stained with DAPI for 15 min. Finally, fluorescent images of the cells were obtained using a Nikon A1Silaser scanning confocal microscope (Nikon Instruments, Inc., Japan). The following antibodies are used for specific molecular markers: Anti-PPP1R15A (10449-1-AP, Proteintech), Anti-DLAT (4A4-B6-C10, Cell Signaling Technology), Anti-EIF2S1/EIF2A (11170-1-AP, Proteintech), CoraLite594–conjugated Goat Anti-Mouse IgG(H + L) (SA00013-3, Proteintech), CoraLite594–conjugated Goat Anti-Rabbit IgG (H + L) (SA00013-4, Proteintech), CoraLite488-conjugated Goat Anti-Mouse IgG(H + L) (SA00013-1, Proteintech), and CoraLite488-conjugated Goat Anti-Rabbit IgG(H + L) (SA00013-2, Proteintech) were used.

### Co-immunoprecipitation

After treatment, the cells were collected by centrifugation using precooled PBS and lysed with NP-40 supplemented with protease inhibitors. Five percent of the cell lysate was removed as the input, and the remaining cells were incubated with protein A/G beads for 2 hours at RT to reduce nonspecific binding. Then, the mixture was equally separated into two Eppendorf tubes and incubated with 5 mg FLAG or IgG antibody overnight at 4 °C. On the second day, protein A/G beads were added, and spin incubation was performed for 3 hours at 4 °C. The sample-loading buffer was added after rinsing. Purified proteins were detected by western blotting. The following antibodies were used for coimmunoblotting: mouse control IgG (AC011; Abclonal) and anti-DDDDK-tag (AE005; Abclonal).

### Tumor xenograft assay

Four-week-old male athymic BALB/c nude mice were purchased from Beijing Vital River Laboratory Animal Technology Co. Ltd.

The mice were subcutaneously injected with tumor cells in PBS after obtaining approval by the Animal Experimentation Ethics Committee of Huazhong University of Science and Technology. During the experiment, mice were randomly divided into the indicated groups (five mice per group), while no blinding was used in the experiments.

To evaluate the anti-tumor effect of elesclomol in vivo, two groups were evaluated: DMSO and Elesclomol. One week after receiving the 1 × 10^6^ tumor cell injection, mice received an intraperitoneal injection of DMSO or elesclomol (50 mg kg^−1^) with 1 μM CuCl_2_ three times a week.

To test whether PPP1R15A knockdown can resist the effect of elesclomol in vivo, four groups of mice were evaluated: Vector, PPP1R15A, Sh-Vector, and Sh-PPP1R15A. Mice received intraperitoneal injection of elesclomol (50 mg kg^−1^) with 1 μM CuCl_2_ three times a week, one week after the initial injection with 2 × 10^6^ tumor cells.

To test the effect of GBZ on elesclomol recovery, we evaluated the following four groups: DMSO, ES, ES + GBZ, and GBZ. Mice received intraperitoneal injection of elesclomol (50 mg kg^−1^) with 1 μM CuCl_2_ alone, GBZ (5 mg kg^−1^) alone or a combination of both three times a week, one week after the initial injection with 1 × 10^6^ tumor cells.

Tumor volume was assessed using the following formula (tumor volume = [length × width^2^]/2). After one month, the mice were euthanized, weighed, and imaged. H&E staining was performed to observe tissue structure. IHC assay was performed to measure Ki67 and PP1R15A expression.

### Chromatin immunoprecipitation

Following the manufacturer’s instructions, a ChIP assay kit (P2078, Beyotime) was used to precipitate the *MYC* enhancer. Briefly, an appropriate amount of formaldehyde was added to the cell culture medium and gently mixed to a final concentration of 1%. Cells were incubated at 37 °C for 10 min to cross-link the target protein with the corresponding genomic DNA. After washing with the glycine solution, the cells were washed with precooled PBS containing 1 mM PMSF and collected. The cells were resuspended in SDS Lysis Buffer containing 1 mM PMSF and lysed on ice before sonication. Finally, the cells were incubated overnight with Protein G Dynabeads pre-incubated with specific antibodies. The primers used for the ChIP-qPCR analysis were as follows:

MYC: 5’-GTCAGCCAATCTTCGCACTT-3’; 5’-TGCCAGAGGAAGCTACTGGT-3’

### Phosphorylated 4D-label free quantitative proteome and transcriptome sequencing

PC-3 cells were transfected with vector and PPP1R15A were determined using phosphorylated 4D-Label free quantitative proteome (Applied Protein Technology, China) and transcriptome sequencing (Novogene, China). Each group included three biological replicates. Sequencing results were obtained in the Supplementary Table.

### Statistical analysis

Data standardization was performed using SPSS Statistics for Windows, Version 25.0. (IBM Corp., Armonk, NY, USA). The other data were analyzed with GraphPad Prism 8.0 (La Jolla, CA, USA) and are shown as the means ± SD. Where appropriate, the *χ*^2^-Test, two-tailed Student’s *t* test were used. *P* < 0.05 was considered statistically significant.

## Results

### Elesclomol induces mitochondrial stress during cuproptosis in cancers

**Fig. 1:**
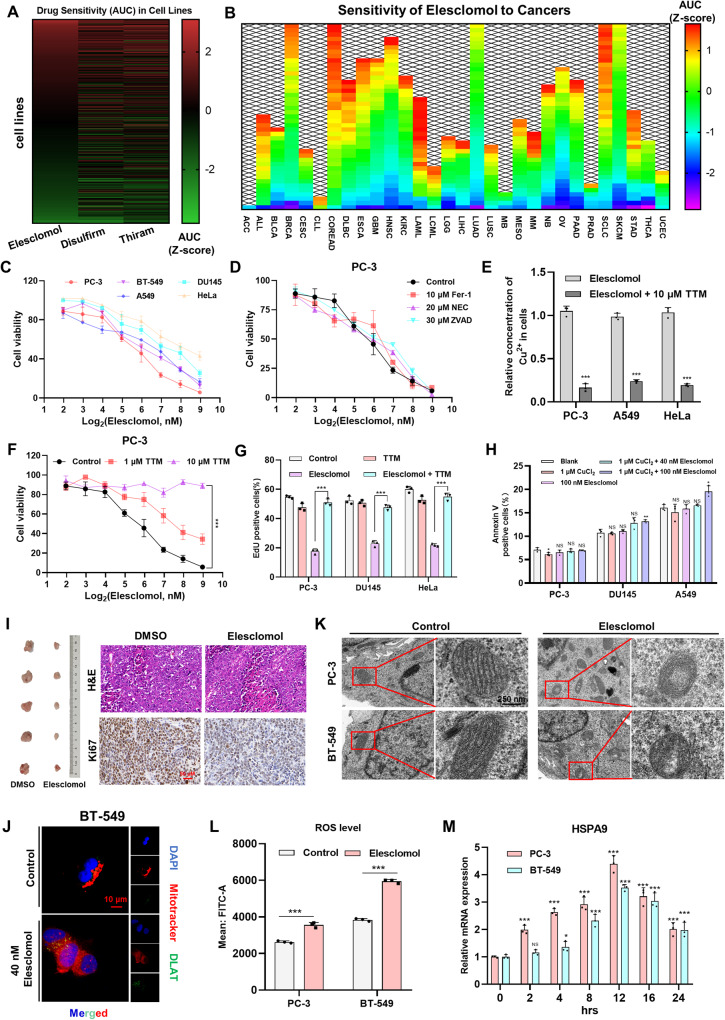
Elesclomol induces mitochondrial stress during cuproptosis in cancers. **A** Heatmap of drug sensitivity of three copper ion carriers to 512 cell lines based on CCLE data. AUC data normalization to compare cell sensitivity to different drugs. The larger the AUC value, the less sensitive the cell line is to the drug. **B** Heatmap of elesclomol sensitivity in different types of cancers based on Genomics of Drug Sensitivity in Cancer data. **C** Viability of cells was assessed by CCK-8 48 hours after pulse treatment of elesclomol. **D** Viability was assessed by CCK-8 after pretreated overnight with 10 μM ferrostatin-1 (Fer-1), 20 μM necrostatin-1(NEC), 30 μM Z-VAD-FMK(Z-VAD), and then treated with elesclomol for 48 hours. **E** Measurement of intracellular copper concentration by Cell Copper (Cu) Colorimetric Assay Kit. The cells were pretreated overnight with 10 μM TTM, and then treated with 100 nM elesclomol for 2 hours. TTM, tetrathiomolybdate. **F** Viability was measured after pretreated overnight with indicated concentrations of TTM and then treated with elesclomol for 48 hours. **G** TTM offset cell proliferation ability. The cells were pretreated overnight with 10 μM TTM and then treated with 100 nM elesclomol for 48 hours. **H** Elesclomol did not induce significant apoptosis. Flow cytometry assay revealed the rate of apoptosis in cells after indicated treatment for 24 hours. **I** Images of excised xenografts acquired using the digital single-lens refex camera. H&E staining was used to observe the tissue structure. IHC assay was performed to measure the expression of Ki67. Scale bars represent 50 μm. **J** DLAT oligomerization was analyzed 24 hours after pulse treatment of 100 nM elesclomol by confocal immunofluorescence imaging (green, DLAT; red, Mitotracker; blue, DAPI). Scale bars represent 10 μm. **K** The morphological changes of mitochondria were detected by TEM 24 hours after pulse treatment of 20 nM elesclomol. Scale bars represent 500 nm. **L** Flow cytometry assay of ROS. The cells were treated with or without 100 nM elesclomol for 48 hours. **M** RT-qPCR was used to determine expression of HSPA9 at indicated time. Cells were treated with 50 nM elesclomol for 2 hours. For elesclomol treatment, except for special instructions, media were supplemented with 1 μM CuCl2, and pulse treatment was incubated for two hours. Data are presented as the means ± SD from three independent experiments. *P < 0.05; **P < 0.01; ***P < 0.001.

To further investigate the specific mechanism of cuproptosis, we obtained the drug sensitivity data of the three copper ion carriers including elesclomol, disulfiram and thiram in 512 tumor cells from the Cancer Cell Line Encyclopedia (CCLE, https://sites.broadinstitute.org/ccle/) (Fig. 1A and Supplementary Table 1). The similarity in the distribution of drug sensitivity among different cell lines for these copper ion carriers may be attributed to their similar structures, which contain two carbon-sulfur double bonds capable of binding copper ions (Supplementary Fig. 1A). Using the Genomics of Drug Sensitivity in Cancer (https://www.cancerrxgene.org/) database, we analyzed the drug sensitivity of elesclomol in various types of cancer (Fig. 1B and Supplementary Table 2). Interestingly, we observed significant heterogeneity in the sensitivity of elesclomol across different cell lines within the same tumor type, suggesting that the response to elesclomol is specific to individual cell lines. Perhaps it can be explained by the inconsistency of mitochondrial respiration intensity of different tumor cells in the same type.

We selected various cell lines from tumors with high incidence in human, including lung, prostate, breast, and cervical cancer, to assess their sensitivity to elesclomol supplemented with CuCl_2_ (Fig. 1C and Supplementary Fig. 1B, C). In order to demonstrate that cuproptosis is a distinct form of programmed cell death, we pre-treated cells with pan-caspase inhibitors (Z-VAD-FMK), ferroptosis inhibitors (Fer-1), and necroptosis inhibitors (NEC) before elesclomol treatment. However, none of these compounds exhibited significant inhibitory effects on the cytotoxic activity of elesclomol (Fig. 1D and Supplementary Fig. 1D). Conversely, tetrathiomolybdate (TTM), the chelator of copper, markedly decreased the copper concentration within the cells (Fig. 1E) and mitigated cell death following elesclomol treatment (Fig. 1F, G and Supplementary Fig. 1E–G). Flow cytometry analysis revealed that elesclomol did not induce apoptosis in cells (Fig. 1H and Supplementary Fig. 1H). In vitro experiment, we demonstrated that elesclomol inhibited the growth of subcutaneous xenografted tumors (Fig. 1I and Supplementary Fig. 1J, K) and did not induce noticeable toxicity in the liver and kidney (Supplementary Fig. 1I).

Furthermore, we confirmed the aggregation of dihydrolipoamide S-acetyltransferase (DLAT) after elesclomol treatment, consistent with the findings of Golub et al. (Fig. 1J and Supplementary Fig. 1L). The use of transmission electron microscopy allowed us to observe structural changes in mitochondria, such as crest breaks and vacuolation, following elesclomol treatment (Fig. 1K). In PC-3 cells, the mitochondrial membrane potential gradually decreased over time after elesclomol treatment, indicating mitochondrial dysfunction (Supplementary Fig. 1M). Similarly, the levels of reactive oxygen species (ROS) and mRNA levels of HSPA9, both indicators of mitochondrial stress, exhibited a similar trend (Fig. 1L, M and Supplementary Fig. 1N). The antioxidant NAC did not prevent elesclomol-induced cell death, indicating that blocking ROS did not offset elesclomol-induced mitochondrial damage (Supplementary Fig. 1O). Therefore, we can conclude that the combination of elesclomol and copper induces mitochondrial dysfunction and stress.

### PPP1R15A knockdown reverses cuproptosis induced by elesclomol

**Fig. 2:**
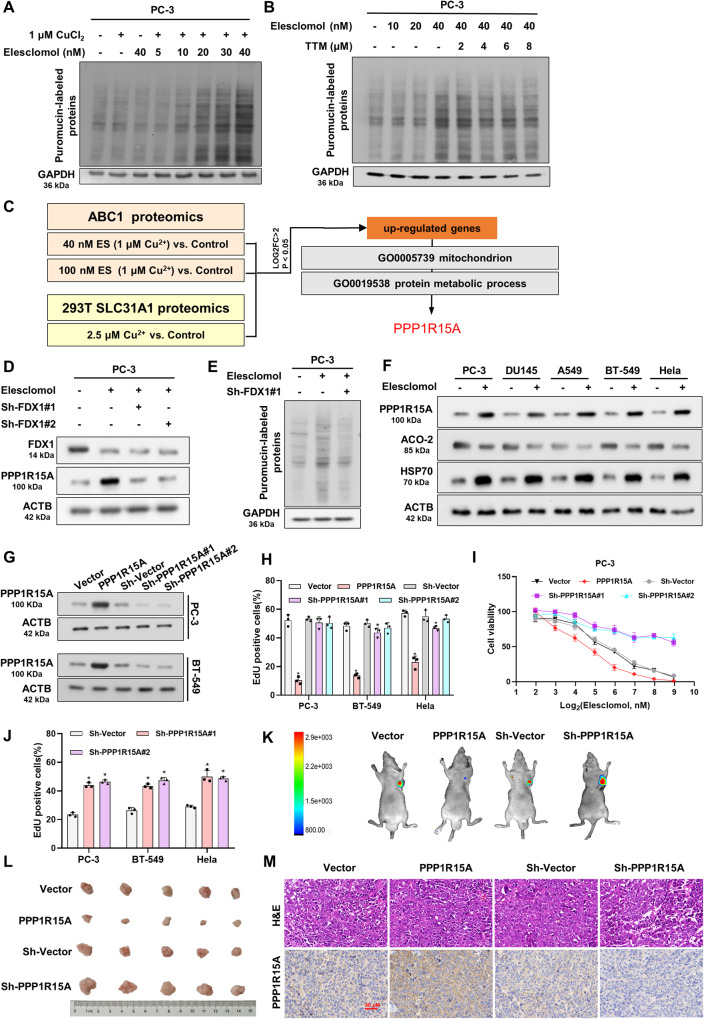
PPP1R15A knockdown reverses cuproptosis induced by elesclomol. **A** Cells were collected 24 hours after treatment of indicated concentrations of elesclomol for 2 hours. Puromycin was added 30 min before cell collection. Puromycin incorporation was detected by western blot to show translation rate. **B** TTM reduced translation rate after elesclomol treatment. The cells were pretreated overnight with indicated concentrations of TTM and then collected 24 hours after 2-hour pulse treatment of indicated concentrations of elesclomol. Puromycin was added 30 min before cell collection. **C** The flowchart for screening out PPP1R15A. **D** Western blot showed FDX1 knockdown reduced the level of PPP1R15A in PC-3 cells 24 hours after pulse treatment of 40 nM elesclomol. **E** Western blot showed FDX1 knockdown reduced translation rate. Protein was collected 24 hours after 2-hour pulse treatment of 40 nM elesclomol. Puromycin was added 30 min before cell collection. **F** Protein content was analyzed in cells 24 hours after pulse treatment of 100 nM elesclomol. **G** Western blot showed the efficiency of PPP1R15A overexpression and knockdown. **H** PPP1R15A overexpression reduced cell viability. **I**, **J** PPP1R15A knockdown attenuated cell death induced by elesclomol. Cell viability was assessed 48 hours after pulse treatment of indicated concentrations of elesclomol (**I**). Cell viability was assessed 24 hours after pulse treatment of 100 nM elesclomol (**J**). **K** Fluorescent images of xenografts in nude mice acquired by an in vivo optical imaging system. **L** Images of excised xenografts acquired using the digital single-lens refex camera. **M** H&E and PPP1R15A immunohistochemical staining of tumors formed by PC-3 cells transfected with vector, PPP1R15A, Sh-Vector and Sh-PPP1R15A. Scale bars represent 50 μm. For elesclomol treatment, except for special instructions, media were supplemented with 1 μM CuCl2, and pulse treatment was incubated for two hours. Data are presented as the means ± SD from three independent experiments. *P < 0.05; **P < 0.01; ***P < 0.001.

To demonstrate the link between increased protein synthesis and proteotoxic stress in cuproptosis, we conducted experiments to evaluate the impact of elesclomol treatment on translation rate. We utilized puromycin, a compound with a structure resembling the 3’ end of aminoacyl-tRNA, which gets incorporated into newly synthesized proteins, allowing us to estimate the rate of protein synthesis [37–39]. In the presence of copper, elesclomol promoted the translation rate in a dose- and time-dependent manner; however, the addition of TTM attenuated this effect (Fig. 2A, B and Supplementary Fig. 2A). To identify genes commonly upregulated in cuproptosis, we analyzed the proteomic results of copper-loaded drugs (ABC1 cells treated with 40 and 100 nM elesclomol) and forced expression of copper transporters (293 T cells overexpressing SLC31A1 supplied with 2.5 µM CuCl2) from Golub et al. Furthermore, considering the association of cuproptosis with mitochondrial and proteotoxic stress, we obtained two gene sets related to mitochondrial (GO0005739) and protein metabolic processes (GO0019538) from the Gene Ontology database. These gene sets were used to further identify the target gene among the co-upregulated genes, leading us to identify PPP1R15A (Fig. 2C and Supplementary Table 3). Meanwhile, we observed NAC could not block the increase of PPP1R15A transcription level induced by elesclomol treatment (Supplementary Fig. 2B). And the increase of PPP1R15A protein level induced by elesclomol treatment was time-dependent (Supplementary Fig. 2C).

We observed that knockdown of FDX1, which is known to cause TCA cycle disorders in cuproptosis, reversed the elesclomol-induced increase in PPP1R15A expression (Fig. 2D and Supplementary Fig. 2D). Additionally, western blotting results demonstrated that FDX1 knockdown reversed the increase in translation rate (Fig. 2E). Similar results were observed with DLAT knockdown, indicating that PPP1R15A is downstream of mitochondrial stress in cuproptosis (Supplementary Fig. 2E). Consistent with previous studies, elesclomol treatment led to an increase in HSP70 and a decrease in ACO-2, an iron-sulfur cluster protein, accompanied by an increase in PPP1R15A expression (Fig. 2F). To investigate the cellular function of PPP1R15A, we created a cell model with PPP1R15A overexpression and knockdown (Fig. 2G). Interestingly, PPP1R15A overexpression significantly impaired cell viability and proliferative capacity, while knockdown had a minimal effect. (Fig. 2H and Supplementary Fig. 2F). This can be attributed to the fact that PPP1R15A is a stress-inducible gene with low basal expression. We further observed that PPP1R15A knockdown reduced cell death induced by elesclomol, whereas PPP1R15A overexpression increased the sensitivity of cells to copper-mediated cell death (Fig. 2I, J and Supplementary Fig. 2G, H). We also observed that PPP1R15A knockdown did not affect the protein level of ACO-2, while the protein level of HSP70 was inhibited after elesclomol treatment. This further supports the notion that PPP1R15A acts downstream of mitochondrial stress and is a key factor in proteotoxic stress (Supplementary Fig. 2I). In subcutaneous xenograft mouse models, PPP1R15A knockdown resulted in significant resistance to elesclomol indicating that PPP1R15A knockdown inhibited elesclomol-induced cell death (Fig. 2K–M and Supplementary Fig. 2J, K).

### PPP1R15A interacts with PP1 to regulate cuproptosis

**Fig. 3:**
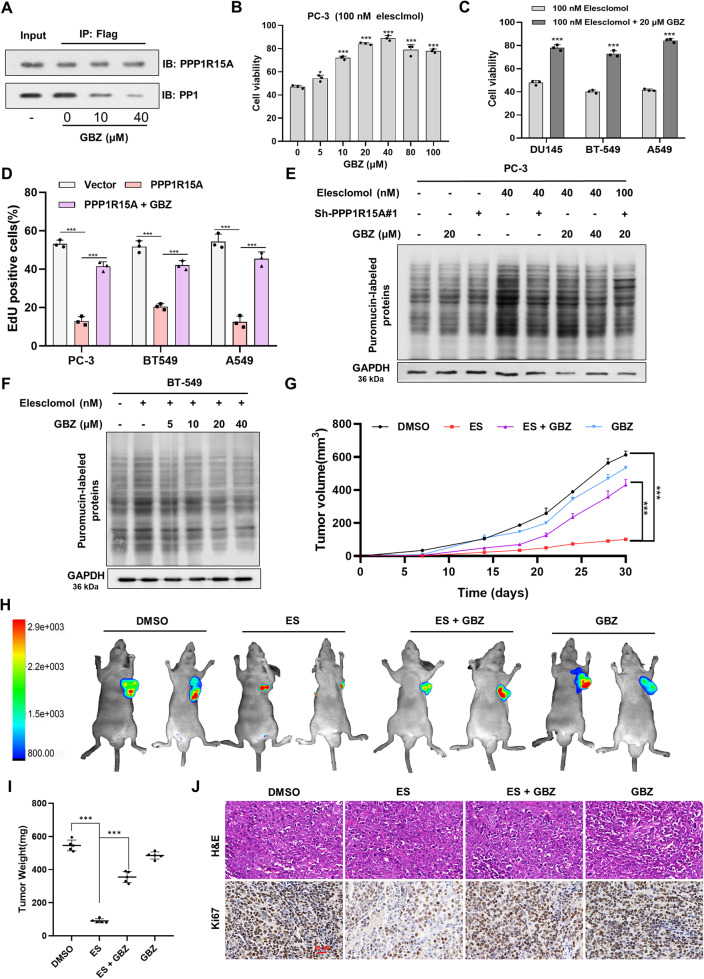
PPP1R15A interacts with PP1 to regulate cuproptosis. **A** Guanabenz (GBZ) prevented PPP1R15A from recruiting PP1 in a dose-dependent manner in 293 T cells transfected with PPP1R15A. **B**, **C** GBZ compensated for cell death induced by elesclomol. Viability was assessed 48 hours after pre-treatment of 100 nM elesclmol for 2 hours and then treatment of indicated concentrations of GBZ (**B**) or 20 μM GBZ (**C**). **D** GBZ offset the impaired cell proliferation caused by PPP1R15A. Viability was measured after treatment of 20 μM GBZ for 48 hours. **E**, **F** GBZ decreased the increase of translation rate by elesclomol. Cells were collected after pre-treatment of 50 nM elesclomol for 2 hours and then treatment of indicated concentrations of GBZ for 24 hours prior to adding puromycin. GBZ, Guanabenz. **G** Tumor volumes were measured during one month. **H** Fluorescent images of xenografts in nude mice acquired by an in vivo optical imaging system. **I** Tumor weights were measured on the day 30. **J** H&E and Ki67 immunohistochemical staining of tumors formed by PC-3 with indicated treatment. Scale bars represent 50 μm. For elesclomol treatment, except for special instructions, media were supplemented with 1 μM CuCl2. Data are presented as the means ± SD from three independent experiments. *P < 0.05; **P < 0.01; ***P < 0.001.

Guanabenz (GBZ), an a2-adrenergic receptor agonist, has been shown to selectively target PPP1R15A^230–674^ containing the PP1 binding site [40]. We confirmed the ability of GBZ to dose-dependently isolate the PPP1R15A-PP1 complex by co-immunoprecipitation experiment (Fig. 3A). GBZ effectively prevented elesclomol-induced cell death indicating that destroying the combination of PPP1R15A and PP1 enhanced cell resistance to cuproptosis (Fig. 3B, C). Furthermore, GBZ rescued the viability of cells overexpressing PPP1R15A (Fig. 3D and Supplementary Fig. 3A). Additionally, PPP1R15A overexpression increased the translation rate, providing confirmation for our hypothesis (Supplementary Fig. 3B). After elesclomol treatment, PPP1R15A knockdown or GBZ effectively reduced the translation rate (Fig. 3E, F and Supplementary Fig. 3C). In vivo, GBZ restricted, to a certain degree, subcutaneous tumor growth under elesclomol treatment. Tumor-bearing nude mice treated with elesclomol and GBZ together showed faster tumor growth rates and larger tumors than elesclomol alone (Fig. 3G–I and Supplementary Fig. 3D). Ki67 staining results showed that GBZ significantly alleviated the impaired proliferation caused by elesclomol (Fig. 3J). In conclusion, PPP1R15A promotes translation rate and reduces cell viability during cuproptosis dependent on interaction with PP1. Based on the above results, we inferred that the phosphorylation events regulated by PPP1R15A-PP1 interaction plays an important role in cuproptosis.

### PPP1R15A promotes translation initiation by regulating the phosphorylation level of EIF2S1 and 4EBP1

To identify potential targets of the PPP1R15A-PP1 complex, we employed co-immunoprecipitation to identify proteins that interact with PPP1R15A. Additionally, we utilized 4D-label-free phosphoproteomics to comprehensively analyze the global protein phosphorylation levels influenced by PPP1R15A regulating the substrate selectivity of PP1 (Supplementary Fig. 4A and Supplementary Table 4). The proteins pulled down by PPP1R15A were found to be enriched in pathways related to the cell cycle, protein stability, and translation (Supplementary Fig. 4B).

**Fig. 4:**
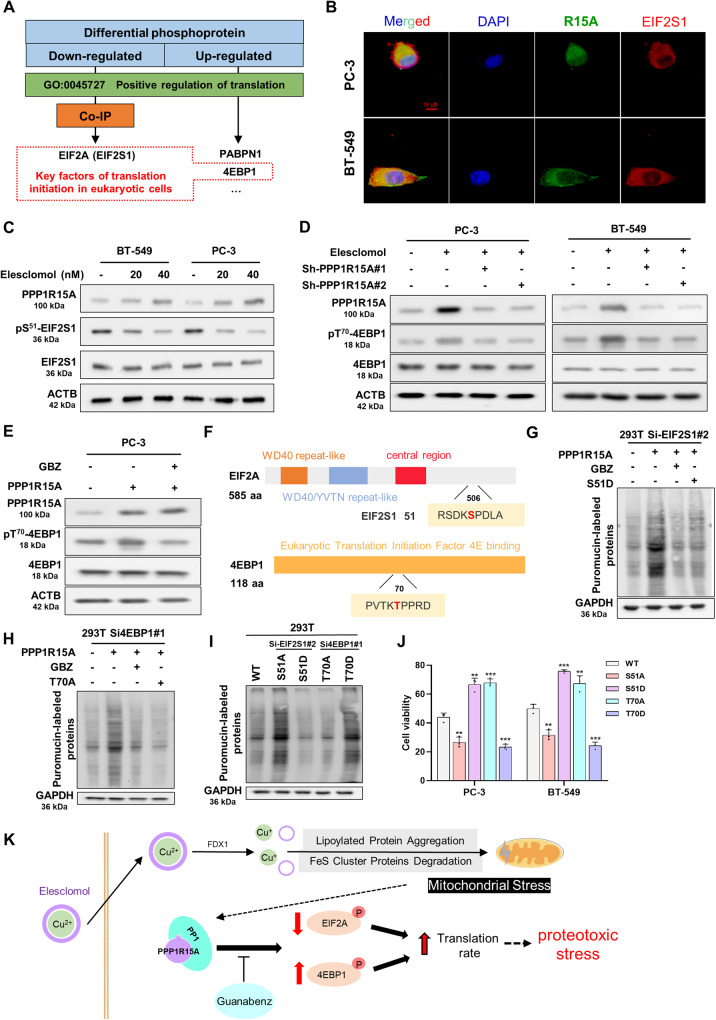
PPP1R15A promotes translation initiation by regulating the phosphorylation level of EIF2S1 and 4EBP1. **A** The flowchart for screening out EIF2S1 and 4EBP1. **B** PPP1R15A and EIF2S1 were co-localized by immunofluorescence. Scale bars represent 10 μm. **C** Western blot showed the phosphorylation level of EIF2S1 at 24 h pulse-treated with indicated concentrations of elesclomol. **D**, **E** PPP1R15A knockdown and GBZ offset the decrease of 4EBP1 phosphorylation level caused by elesclomol. Western blot showed the phosphorylation level of 4EBP1 at 48 h in cells transfected with Sh-PPP1R15A, pulse-treated with 50 nM elesclomol or treated with 20 μM GBZ for 48 hours. **F** Diagram of EIF2S1 and 4EBP1 phosphorylation site mutation. **G** S51D decreased rate of protein synthesis in cells. S51D, mutation of EIF2S1 S51 to the phosphomimetic amino acid aspartic acid. 293 T cells were transfected with Si-EIF2S1#2. **H**, **I** Rate of translation in cells with indicated treatment. For GBZ treatment, the cells were treated with 20 μM GBZ for 48 hours. S51A, mutation of EIF2S1 S51 to the non-phosphorylatable amino acid alanine; T70A, mutation of 4EBP1 T70 to the non-phosphorylatable amino acid alanine; T70D, mutation of 4EBP1 T70 to the phosphomimetic amino acid aspartic acid. 293 T cells were transfected with indicated siRNA. **J** Cell viability transfected with mutation of EIF2S1 and 4EBP1 were assessed 24 hours after pulse treatment of 50 nM elesclomol for 2 hours. **K** Mechanism map of increased translation rate induced by elesclomol. For elesclomol treatment, media were supplemented with 1 μM CuCl2. Data are presented as the means ± SD from three independent experiments. *P < 0.05; **P < 0.01; ***P < 0.001.

To identify potential targets that promote translation, we screened the upregulated and downregulated phosphorylated proteins using the “positive regulation of translation” gene set (GO:0045727) from the Gene Ontology database. Our focus was on key factors involved in translation initiation, specifically EIF2S1 and 4EBP1 (Fig. 4A). EIF2S1 is a well-known target of PPP1R15A, and previous studies have demonstrated that PPP1R15A dephosphorylates EIF2S1 at S51, leading to the reversal of global translational arrest [23, 41, 42]. Confocal microscopy confirmed the colocalization of EIF2S1 and PPP1R15A in cells, and immunoblotting confirmed that PPP1R15A induced by elesclomol decreased the expression of pS51-EIF2S1 (Fig. 4B, C). Furthermore, western blotting revealed that PPP1R15A knockdown inhibited the increase in pT70-4EBP1 caused by elesclomol, indicating that PPP1R15A is necessary for elesclomol-induced changes in 4EBP1 phosphorylation (Fig. 4D). Treatment with GBZ reversed the increase in pT70-4EBP1 mediated by PPP1R15A overexpression, suggesting that PPP1R15A alters PP1 selectivity to maintain 4EBP1 phosphorylation (Fig. 4E and Supplementary Fig. 4C).

To further validate the role of phosphorylation sites identified through phosphoproteomics, we constructed a mutation of EIF2S1 S51 in the non-phosphorylatable amino acid alanine (S51A) and the phosphomimetic amino acid aspartic acid (S51D) and a mutation of 4EBP1 T70 in the non-phosphorylatable amino acid alanine (T70A) and the phosphomimetic amino acid aspartic acid (T70D) (Fig. 4F). In order to eliminate the influence of endogenous EIF2S1 and 4EBP1, we verified the knockdown efficiency of the corresponding siRNAs, and finally selected Si-EIF2S1#2 and Si-4EBP1#1 for subsequent functional experiments (Supplementary Fig. 4D). In 293 T cells, the S51D mutation attenuated the increase in translation rate induced by PPP1R15A (Fig. 4G), while the T70A mutation rendered PPP1R15A ineffective in regulating the translation rate. (Fig. 4H). Interestingly, dephosphorylation of EIF2S1 and phosphorylation of 4EBP1 increased the translation rate and decreased cell viability (Fig. 4I, J).

As shown in the mechanism diagram, elesclomol facilitated the entry of copper ions into cells, inducing PPP1R15A expression, which in turn decreased pS51-EIF2S1 and increased pT70-4EBP1 through its interaction with PP1. This led to an increased translation rate and ultimately drove cells to undergo proteotoxic stress, potentially resulting in cell death (Fig. 4K).

### PPP1R15 A promotes MYC silencing and G2M phase arrest

**Fig. 5:**
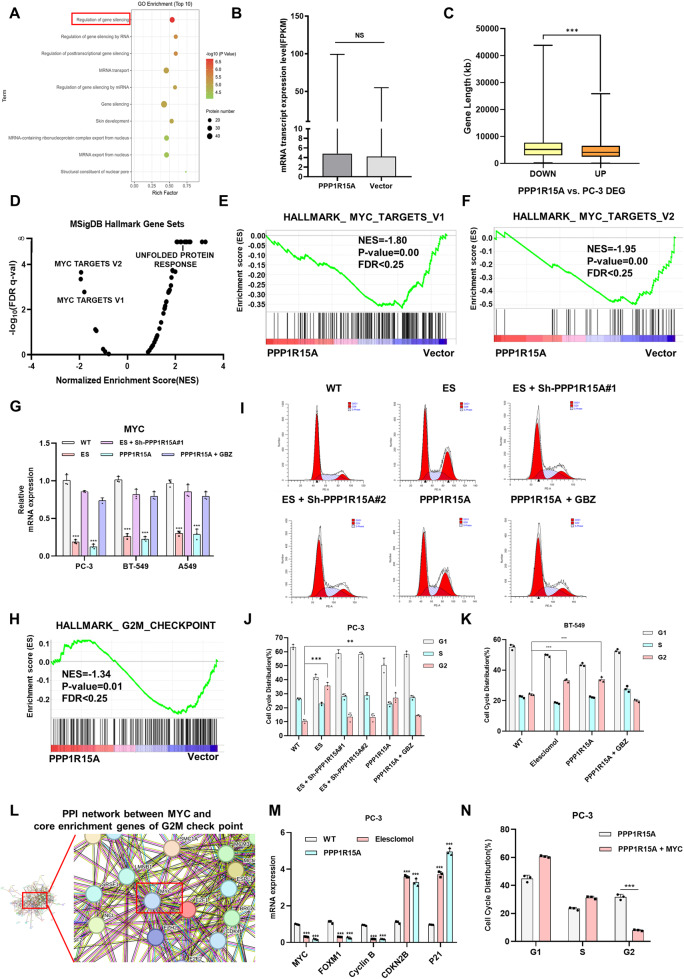
PPP1R15A promotes *MYC* silencing and G2M phase arrest. **A** GO functional enrichment bubble diagram of differentially expressed phosphorylated peptides in PPP1R15A vs. Vector group under biological process classification. **B** Comparation of mRNA transcription expression level (FPKM) in PC-3 transfected with vector and PPP1R15A. **C** Gene lengths of up and downregulated by PPP1R15A (Log2FC ≥ 1 and *P* ≤ 0.05). **D** GSEA analysis of transcriptome sequencing data of vector and PPP1R15A based on Hallmark genes sets. **E**, **F** The genes in the PPP1R15A group were negatively enriched to the MYC targets. **G** Relative mRNA expression level of MYC was assessed by RT-qPCR. **H** The genes in the PPP1R15A group were negatively enriched to the G2M checkpoint. **I** Flow cytometry assay revealed cell cycle in PC-3 under indicated condition. ES, elesclomol. **J** Quantitation of Fig. 5**I**. **K** Quantitation of cell cycle in BT-549 under indicated condition. **L** PPI network between MYC and core enrichment genes of G2M checkpoint in GSEA. **M** Relative mRNA expression level of MYC and its targets was assessed by RT-qPCR. **N** Forced expression of MYC alleviated G2M phase arrest in PPP1R15A overexpression PC-3. For elesclomol treatment, media were supplemented with 1 μM CuCl2. Data are presented as the means ± SD from independent experiments. *P < 0.05; **P < 0.01; ***P < 0.001.

The Gene Ontology enrichment analysis of differentially phosphorylated proteins in phosphoproteomics revealed a relationship between PPP1R15 A and gene silencing (Fig. 5A). Gene silencing describes the process carried out at the cellular level that results in either long-term transcriptional repression via action on chromatin structure or RNA mediated, post-transcriptional repression of gene expression. To understand the mechanism of PPP1R15A-induced gene silencing and resolve the contradiction between reduced transcription and increased translation, we conducted transcriptome sequencing (Supplementary Fig. 5A). We adopted the method described by Vihandha et al. to evaluate whether the transcript levels were reduced due to gene silencing induced by PPP1R15A [43]. However, the level of fragments per kilobase of transcript per million mapped reads (FPKM) did not show significant differences between PC-3 cells transfected with or without PPP1R15A (Fig. 5B). Interestingly, the significantly upregulated genes (Log2FC > 1, FDR < 0.25) in the PPP1R15A group were relatively short in length compared to the vector group, which is consistent with the stress state (Fig. 5C). This discrepancy can be explained by the fact that highly expressed and rapidly regulated genes tend to have fewer introns and are shorter than average in mammalian cells [43, 44]. This suggested that it is not the entire transcriptome that is silenced, but rather specific genes. To identify these silenced genes, we performed Gene Set Enrichment Analysis (GSEA) using MSigDB Hallmark Gene Sets (Fig. 5D). The downregulated genes were found to be highly enriched in MYC targets (version 1 and 2) (Fig. 5E, F), while the response to unfolded and misfolded proteins was positively enriched in the PPP1R15A group (Supplementary Fig. 5B). We confirmed that MYC mRNA levels were inhibited by elesclomol and PPP1R15A (Fig. 5G).

Considering that the enrichment results mentioned above, which include cell cycle-related pathways (Supplementary Fig. 4C), and the negative enrichment of the G2M checkpoint in the control group (Fig. 5H), we examined cell cycle under indicated treatment. Flow cytometry analysis revealed that both elesclomol and PPP1R15A induced G2M phase arrest, which could be rescued by PPP1R15A knockdown or GBZ treatment (Fig. 5I–K and Supplementary Fig. 5C). These results indicated that PPP1R15A induce G2M phase arrest interacting with PP1 during cuproptosis. To further validate that PPP1R15A-induced G2M phase arrest depends on the silencing of MYC and its targets, we investigated the relationship between MYC, a regulator of cell cycle, and the core enrichment genes of the G2M checkpoint using the STRING website (Fig. 5L). We examined the expression of MYC and target genes associated with G2M phase arrest [45, 46] (Fig. 5M). Additionally, the rescue experimental results were consistent with our hypothesis that PPP1R15A-induced G2M phase arrest is dependent on the silencing of MYC (Fig. 5N and Supplementary Fig. 5D–G).

### PPP1R15A plays a role in silencing MYC by regulating the activity of lysine methyltransferase and reducing H3K4me levels

**Fig. 6:**
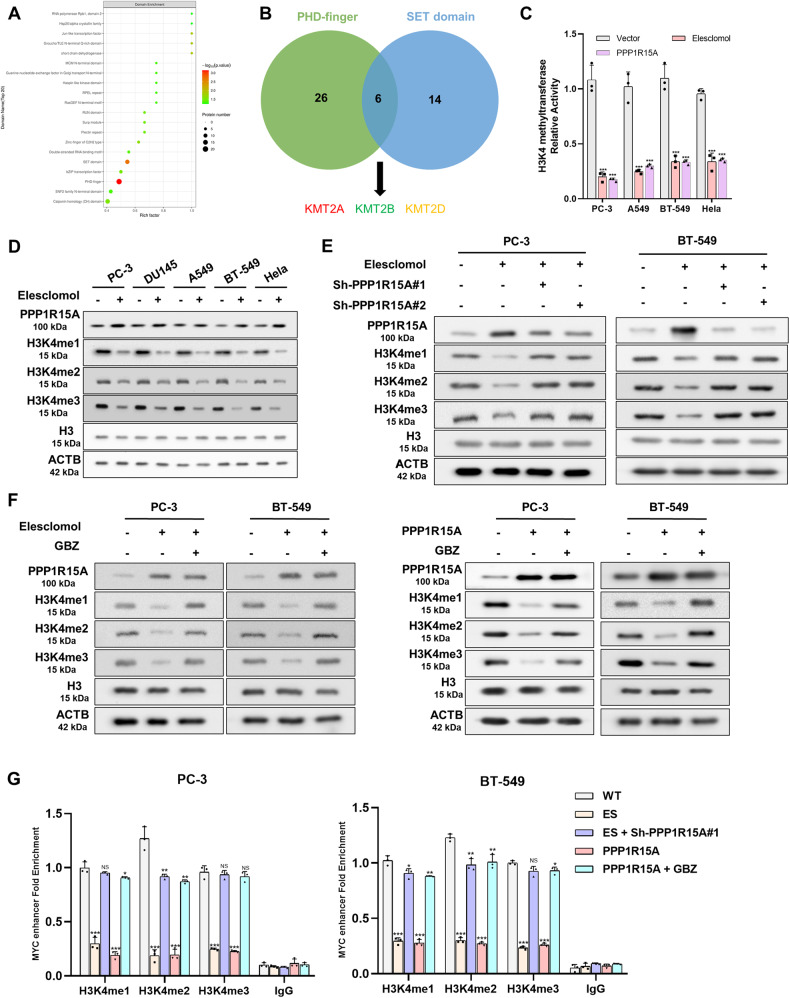
PPP1R15A plays a role in silencing MYC by regulating the activity of lysine methyltransferase and reducing H3K4me levels. **A** Diagram of domain enrichment analysis of proteins of the differentially expressed modified peptides in the PPP1R15A vs. Vector group. **B** Venn diagram of the intersection of PHD-finger and SET domain. Red, increase of phosphorylation level; green, down; yellow, up or down in different sites. **C** Elesclomol and PPP1R15A reduced H3K4 methylation level in cells using Histone H3 (K4) Methyltransferase Activity Quantification Assay Kit. **D** Western blot showed that elesclomol induced decrease of H3K4 methylation. **E**, **F** PPP1R15A knockdown (**E**) and GBZ (**F**) offset the effect of elesclomol on H3K4 methylation. Proteins were collected 24 hours after 2-hour pulse treatment of 100 nM elesclomol. **G** ChlP-qPCR for H3K4me1, H3K4me2, H3K4me3 and IgG control with indicated treatment. Data are presented as the means ± SD from independent experiments. *P < 0.05; **P < 0.01; ***P < 0.001.

To investigate the underlying mechanism of PPP1R15A-mediated MYC silencing, we examined the domain enrichment results related to histone modification (Fig. 6A). Among the significantly enriched domains in the differentiated phosphoproteins, we identified KMT2A, KMT2B, and KMT2D, which are associated with H3K4me [33] (Fig. 6B). Due to the complex phosphorylation patterns at different sites of these lysine methyltransferases, we decided to assess the overall methyltransferase activity of H3K4. Our findings revealed that elesclomol treatment or PPP1R15A overexpression reduces the methyltransferase activity of H3K4 in cells (Fig. 6C). Western blot analysis demonstrated that elesclomol treatment led to a decrease in H3K4me1/2/3 levels (Fig. 6D). Moreover, the silencing of PPP1R15A and treatment with GBZ reversed the epigenetic regulation of H3K4 (Fig. 6E, F). The reduction in H3K4me levels facilitated gene silencing, which corroborated our previous observations. Given that H3K4me is a marker of active chromatin primarily found in enhancers, we performed ChIP-qPCR experiments to confirm that H3K4me promotes MYC enhancer activation and transcriptional output [28, 36]. Consistent with previous studies, H3K4me promoted the expression of MYC (Fig. 6G).

These results indicated that the epigenetic regulation of H3K4 by elesclomol is dependent on the interaction between PPP1R15A and PP1. Additionally, PPP1R15A promotes MYC silencing through histone modification.

### Cuproptosis mechanisms are shared in the model of copper homeostasis dysregulation

**Fig. 7:**
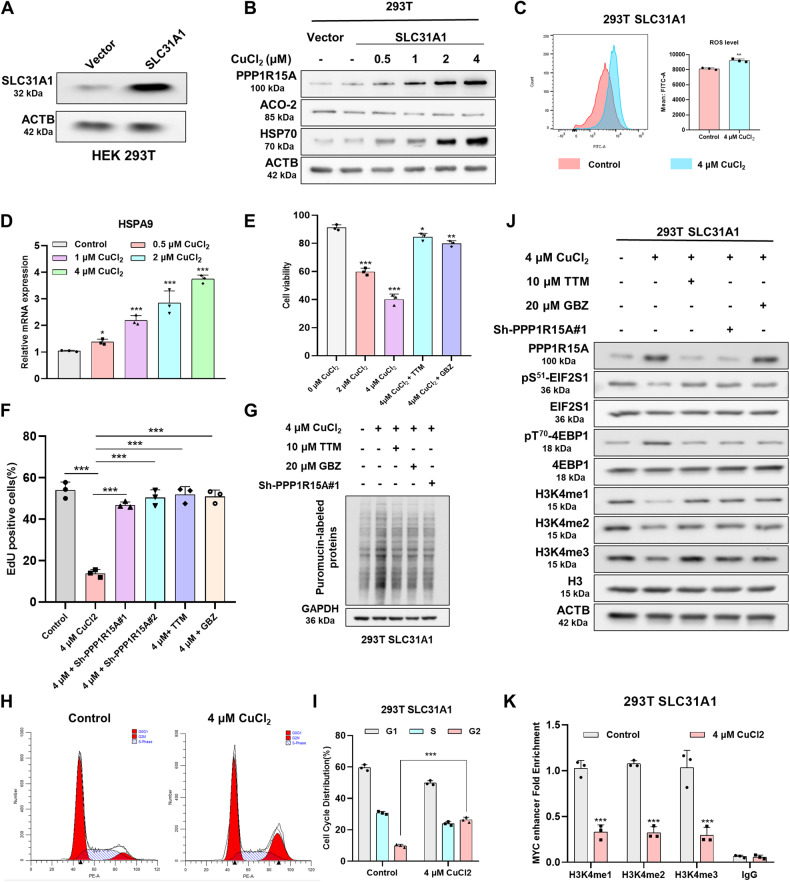
Cuproptosis mechanisms are shared in the model of copper homeostasis dysregulation. **A** Protein level of SLC31A1 was assessed by western blot. **B** Protein content was analyzed and SLC31A1 overexpression 293 T cells 24 hours after treatment of supplementation with indicated concentrations of CuCl2. **C** Flow cytometry assay of ROS. The cells were treated with or without 4 μM CuCl_2_ for 24 hours. **D** RT-qPCR was used to determine expression of HSPA9 at indicated treatment. **E**, **F** Viability was measured by CCK-8 (**C**) and EDU (**D**) in 293 T cells and SLC31A1 overexpression 293 T cells under indicated treatment. **G** Protein content was analyzed and SLC31A1 overexpression 293 T cells with indicated treatment for 24 hours. **H**, **I** Cell cycle of SLC31A1 overexpression 293 T cells 24 hours after treatment of supplementation with or without CuCl2. **J** Protein content was analyzed in SLC31A1 overexpression 293 T cells 24 hours after indicated treatment. For treatment of TTM and GBZ, the cells were treated with 10 μM TTM or 20 μM GBZ for 24 hours and then collected. **K** ChlP-qPCR for H3K4me1, H3K4me2, H3K4me3 and IgG control in SLC31A1 overexpression 293 T cells 24 hours after treatment of supplementation with or without CuCl2. Data are presented as the means ± SD from independent experiments. *P < 0.05; **P < 0.01; ***P < 0.001.

To demonstrate the connection between cuproptosis and imbalanced copper homeostasis, we established a cell model overexpressing solute carrier family 31 member 1 (SLC31A1) to eliminate the drug-specific effects of elesclomol (Fig. 7A). SLC31A1 is a high-affinity, saturable copper transporter in the cell membrane involved in copper uptake. By increasing the copper concentration in the media, we observed an elevation in PPP1R15A and HSP70 levels, accompanied by a decrease in ACO-2 levels (Fig. 7B). The protein level of PPP1R15A was significantly increased in PC-3 cells and BT-549 cells overexpressing SLC31A1 supplemented with copper ions, which indicated that PPP1R15A was a necessary regulator of cell death after copper ions entered the cells (Supplementary Fig. 6A, B). The mitochondrial membrane potential decreased in 293 T cells overexpressing SLC31A1 supplemented with copper ions, while the levels of ROS and HSPA9 increased. This indicates that copper ions could induce mitochondrial dysfunction alone (Fig. 7C, D and Supplementary Fig. 6C). Treatment with TTM, GBZ, or PPP1R15A knockdown rescued cell viability and proliferation (Fig. 7E, F and Supplementary Fig. 6D). In addition, treatment with TTM, GBZ, or PPP1R15A knockdown deduced translation rate leading to alleviate copper-induced proteotoxic stress (Fig. 7G). In the SLC31A1-overexpressing 293 T cell model, copper also decreased *MYC* level and induced G2M phase arrest (Fig. 7H, I and Supplementary Fig. 6E). Our western blotting results revealed that the entry of copper ions into the cells led to an upregulation of PPP1R15A, which subsequently regulated the phosphorylation of EIF2S1 and 4EBP1, as well as the epigenetic modification of H3K4 (Fig. 7J). Furthermore, ChIP-qPCR experiment confirmed that copper reduces the activity of MYC enhancer (Fig. 7K).

**Fig. 8:**
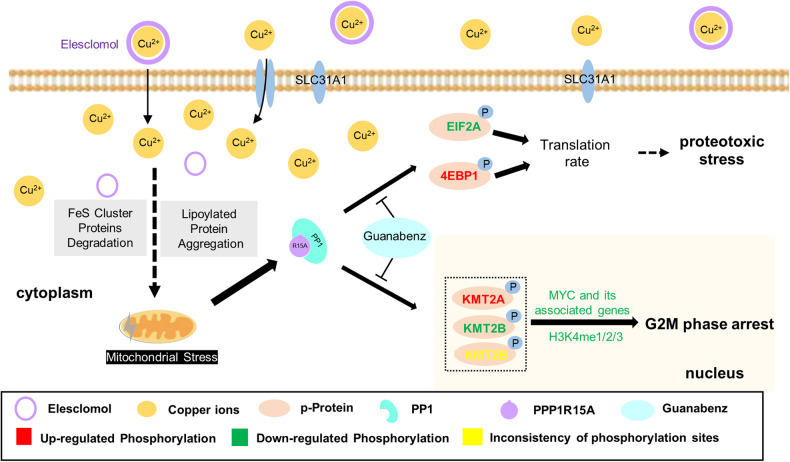
Mechanism diagram of shared copper death caused by PPP1R15A. The increase of intracellular copper ions induced mitochondrial damage and then induced the expression of PPP1R15A. PPP1R15A enhanced translation initiation, thereby promoting proteotoxic stress by regulating the phosphorylation of EIF2S1 and 4EBP1. PPP1R15A reduced H3K4me levels, resulting in the suppression of MYC and its associated genes by modulating the phosphorylation of KMT2A/B/D, which subsequently induced G2M phase arrest.

We identified that PPP1R15A was elevated in both the copper-loaded drug model and the copper homeostasis disorder model. This elevated PPP1R15A promoted translation initiation, inducing proteotoxic stress, and silenced MYC through histone methylation modification, leading to G2M phase arrest (Fig. 8).

## Discussion

In the past decade, a growing body of evidence has highlighted the role of essential trace metals, such as zinc, iron, and copper, in exerting toxicity on cells and ultimately leading to cell death. Studies in yeast and mammalian cells have specifically linked copper toxicity to mitochondrial dysfunction [47]. Cuproptosis, a form of cell death, promotes the degradation of Fe-S cluster proteins and induces proteotoxic stress by stimulating the aggregation of lipoylated proteins, ultimately resulting in cell death [12]. Our research revealed significantly elevated levels of PPP1R15A, a protein associated with mitochondrial and protein metabolism. PPP1R15A not only contributes to proteotoxic stress but also plays a role in MYC silencing during cuproptosis. While previous studies focused on the dephosphorylation effects of PPP1R15A, our experiments demonstrated the importance of phosphorylation events as well.

Mitochondria, the powerhouses of cells responsible for energy synthesis and supply, are susceptible to various sources of stress, including loss of membrane integrity, disruption of membrane potential, and metabolic dysfunction. A When cells encounter stress, a common response is to reduce proliferation, metabolic activity, and shut down energy-demanding biological processes. Protein synthesis, being the most energy-consuming process in cells, is particularly affected [48]. Phosphorylation of EIF2S1, which suppresses global translation, is a common mechanism employed by cells to cope with mitochondrial stress [49]. During cuproptosis, mitochondria sustain irreversible damage, leading to continuous induction of PPP1R15A, which dephosphorylates EIF2S1 while phosphorylating 4EBP1, thereby promoting translation initiation. This excessive protein synthesis overwhelms the cell’s capacity to maintain protein homeostasis resulting in proteotoxic stress. The close relationship between copper metabolism disorders and neurological diseases, such as Wilson’s disease, Alzheimer’s disease, Parkinson’s disease, and amyotrophic lateral sclerosis which are often associated with protein deposition, has garnered significant attention from researchers [1, 50]. The intricate interplay between copper, mitochondria, and protein homeostasis constitutes a complex regulatory network that requires further elucidation.

Histone modification is a crucial epigenetic mechanism that regulates chromatin dynamics and gene expression, playing a significant role in tumor occurrence and development. Due to the complex phosphorylation patterns of KMT2A, KMT2B, and KMT2D at different sites, we chose to measure the overall methyltransferase activity of H3K4. The specific impact of site-specific phosphorylation of these methyltransferases on H3K4me is still unclear. However, it has been established that H3K4me can regulate MYC expression. MYC is a well-known oncogene and a key regulator of various genes involved in cell proliferation and metabolic processes. Several studies indicate that MYC proteins play a specific role during the G2 phase. For instance, the transcription factor FOXM1, a critical target of MYC-induced cell growth, is involved in G2 progression and is consistent with the direct target cyclin B1, which promotes mitosis [45]. Moreover, MYC can inhibit CDK inhibitors P21 and CDKN2B to restore cell cycle arrest [46]. Polo-like Kinase-1 also associates with MYC and controls its stability and possibly its transcriptional functions during G2 phase and mitosis [51]. However, in a different experimental setup, such as cells that have been exposed to DNA damage (an anti-mitogenic stimulus), transcriptional responses to MYC are very different [52]. Hence, the identity of the genes regulated by MYC appears to be largely determined by the experimental conditions and cell types used [45]. A comprehensive and detailed identification of the specific targets of MYC in cuproptosis will be a complicated and giant project, but it will also help us to further understand the specific mechanism. During cuproptosis, MYC silencing induces G2M phase arrest, and the underlying selective regulatory mechanism warrants further investigation. Copper has been reported to induce cell cycle arrest by generating reactive oxygen species and interfering with cell metabolism [53, 54]. Our findings provide new insights into the regulation of the cell cycle by copper.

Based on the results and discussions above, we acknowledge that the mechanism of cuproptosis is complex and not fully understood. However, PPP1R15A has demonstrated strong potential in inducing cell death. Our database analysis reveals that copper ionophore drugs exhibit similar sensitivities across different cell types, suggesting that the underlying mechanisms may be similar or overlapping. The identification of a suitable marker is essential to determine whether patients can benefit from copper ion carriers. Future studies will focus on designing carrier drugs and targeting tumor cells more effectively.

### Supplementary information

Supplementary Figures

Supplementary Tables

Reproducibility checklist

Western Blotting

## Data Availability

The data underlying this article are available in the article and in its online supplementary material.
