# Supplementary material for: Protein phosphatase 1 regulatory subunit 15 A promotes translation initiation and induces G2M phase arrest during cuproptosis in cancers
Source: Cell Death Dis. 2024 Feb 16;15(2):149. doi: 10.1038/s41419-024-06489-w (PMC10873343; doi:10.1038/s41419-024-06489-w)
Supplement: Supplementary file 1 — Supplementary Figures [file 41419_2024_6489_MOESM1_ESM.pdf]

Supplementary Figure

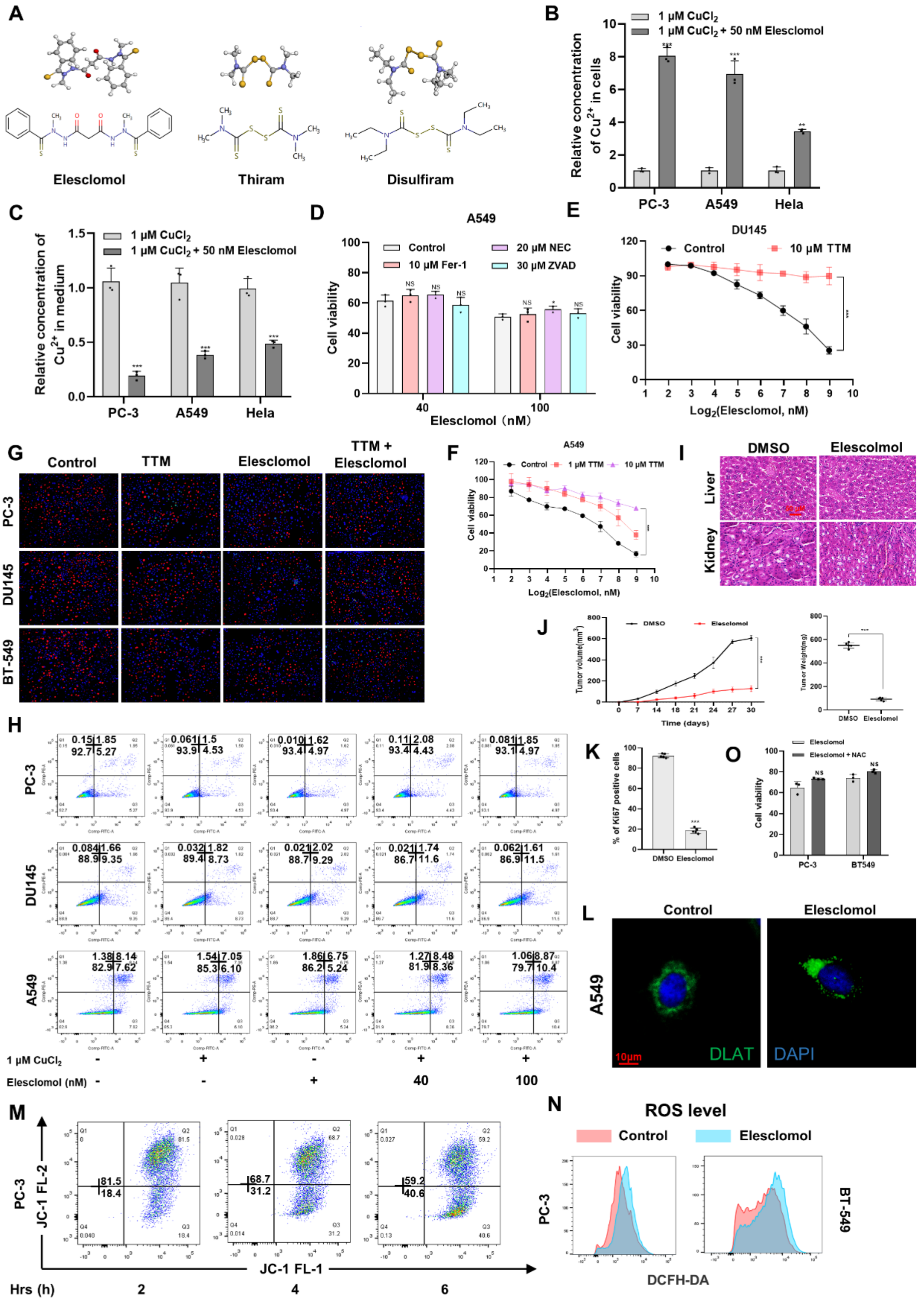

Supplementary Figure 1. Elesclomol induces mitochondrial stress during cuproptosis in cancers

(A) Drug structure of three copper ion carriers form DrugCentral.

(B and C) Measurement of intracellular (B) or medium (C) copper concentration by Copper (Cu) Colorimetric Assay Kit. The cells were treated with 50 nM elesclomol for 2 hours supplemented with or without 1 μM $CuCl_2$.

(D) Viability was assessed after pretreated overnight with 10 μM Fer-1, 20 μM NEC, 30 μM ZVAD and then treated with indicated concentrations of elesclomol for 48 hours.

(E and F) TTM rescued cell viability after treatment of elesclomol. The cells were pretreated overnight with TTM and then treated with indicated concentrations of elesclomol for 48 hours.

(G) Representative images of Fig. 1G.

(H) Representative images of Fig. 1 H.

(I) H&E staining showed that the treatment of elesclomol did not significantly change the structure of liver and kidney. Scale bars represent 50 μm.

(J) Tumor volumes were measured during one month. Tumor weights were measured on the day 30.

(K) Quantitation of Fig. 1I.

(L) DLAT oligomerization was analyzed 24 hours after 2-hour pulse treatment of 100 nM elesclomol by confocal immunofluorescence imaging (green, DLAT; blue, DAPI). Scale bars represent 10 μm.

(M) Flow cytometry assay revealed elesclomol decreased mitochondrial membrane potential in a time-dependent manner. PC-3 cells were treated with 20 nM elesclomol for 2 hours.

(N) Representative images of Fig. 1L.

(O) Viability was assessed after treated with 5 mM NAC and 20 nM elesclomol for 48 hours.

Data are presented as the means ± SD from independent experiments. *, $P < 0.05$; **, $P < 0.01$; ***, $P < 0.001$.

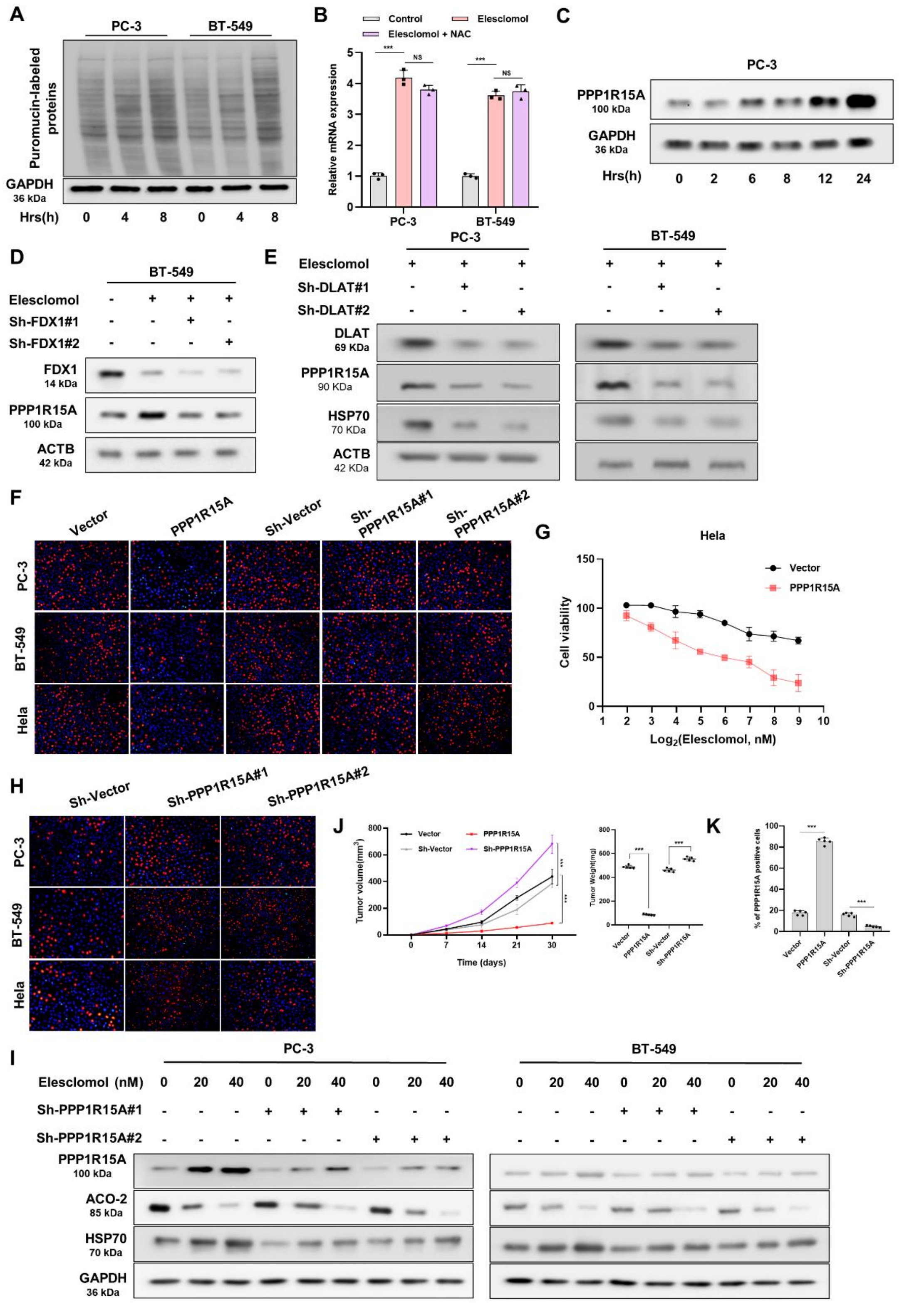

Supplementary Figure 2. PPP1R15A knockdown reverses cuproptosis induced by elesclomol

(A) Elesclomol induced the increase of translation rate in a time-dependent manner.

The cells were treated with 50 nM elesclolmol for 2 hours.

(B) Relative mRNA expression level of PPP1R15A under indicated treatment. NAC, 5 mM; elesclomol, 20 nM.

(C) Elesclomol promotes the expression of PPP1R15A in a time-dependent manner. Western blot was performed to examine the expression of PPP1R15A after 2-hour pulse treatmentof 20 nM elesclomol.

(D) Western blot showed FDX1 knockdown reduced the level of PPP1R15A in BT-549 cells 24 hours after pulse treatment of 40 nM elesclomol.

(E) Western blot showed DLAT knockdown reduced cuproptosis.

(F) Representative images of Fig. 2H.

(G) PPP1R15A overexpression promoted cell death induced by elesclomol. Cell viability was assessed 48 hours after pulse treatment of indicated concentrations of elesclomol

(H) Representative images of Fig. 2J.

(I) Western blot showed PPP1R15A knockdown reduced expression of HSP70. Cells were collected 24 hours after treatment of indicated concentrations elesclomol for 2 hours.

(J) Tumor volumes were measured during one month. Tumor weights were measured on the day 30.

(K) Quantitation of Fig. 2M.

Data are presented as the means ± SD from independent experiments. *, $P < 0.05$; **, $P < 0.01$; ***, $P < 0.001$.

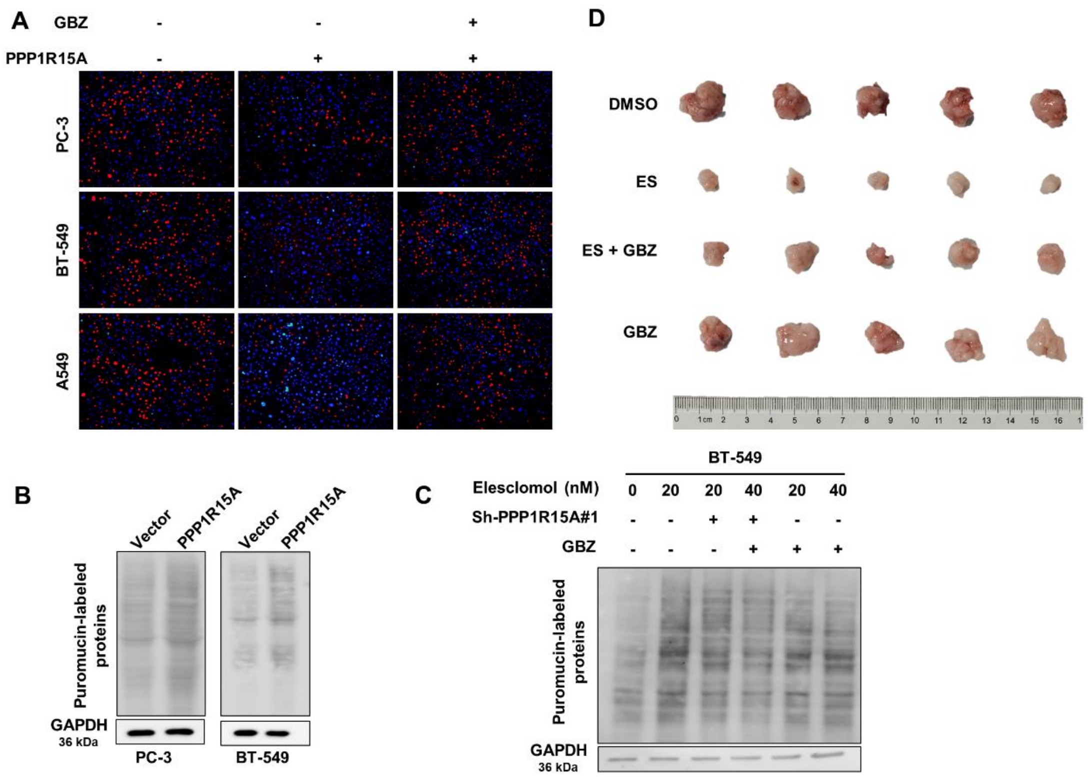

Supplementary Figure 3. PPP1R15A interacts with PP1 to regulate cuproptosis

(A) Representative images of Fig. 3D.

(B) PPP1R15A overexpression promoted translation rate.

(C) PPP1R15A knockdown attenuated the increase translation rate caused by elesclomol. Puromycin was added 30min before cell collection.

(D) Images of excised xenografts acquired using the digital single-lens refex camera.

Data are presented as the means ± SD from independent experiments. *, $P < 0.05$; **, $P < 0.01$; ***, $P < 0.001$.

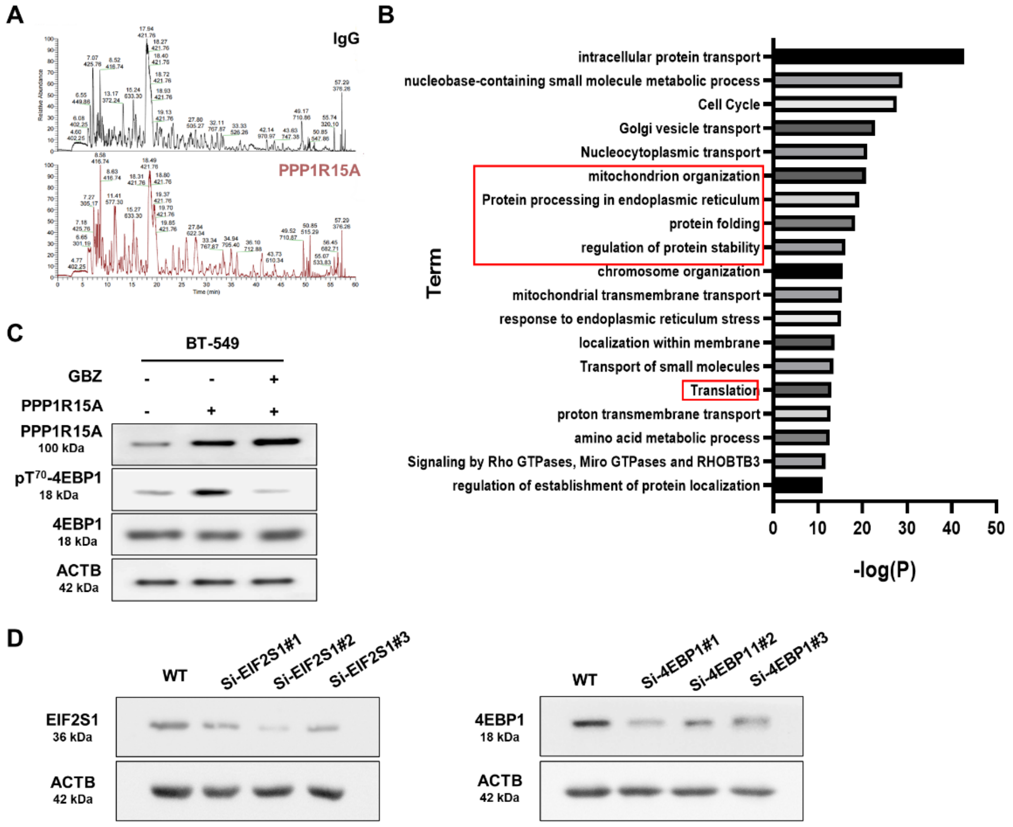

Supplementary Figure 4. PPP1R15A promotes translation initiation by regulating the phosphorylation level of EIF2S1 and 4EBP1

(A) Base peak of proteins pulled down by IgG and FLAG-PPP1R15A. Statistic results of phosphoproteome.

(B) Functional enrichment of proteins pulled down by FLAG using Metascape.

(C) GBZ offset the decrease of 4EBP1 phosphorylation level caused by PPP1R15A in BT-549 cells.

(D) The knockdown efficiency of the siEIF2S1 and si4EBP1.

Data are presented as the means ± SD from independent experiments. *, $P < 0.05$; **, $P < 0.01$; ***, $P < 0.001$.

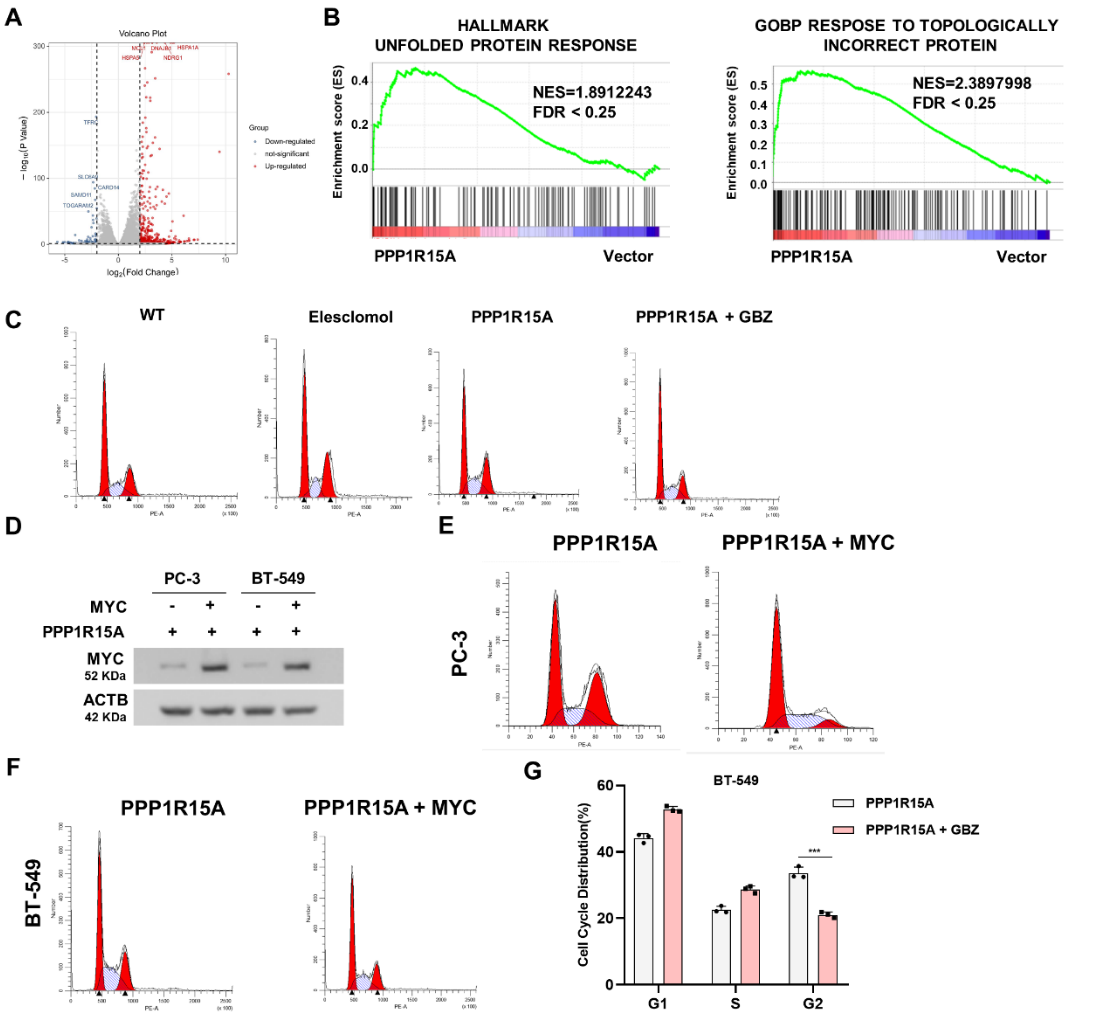

Supplementary Figure 5. PPP1R15A promotes *MYC* silencing and G2M phase arrest

(A) Volcano plot of differential genes in PPP1R15A vs. Vector group.

(B) Representative images of GSEA analysis based on KEGG genes sets.

(C) Representative images of Fig. 5K.

(D) The overexpression efficiency of MYC was detected by immunoblotting.

(E) Representative images of Fig. 5N.

(F) Forced expression of MYC alleviated G2M phase arrest in PPP1R15A overexpression BT-549.

(G) Quantitation of supplementary Fig. 5F.

Data are presented as the means ± SD from three independent experiments. *, P < 0.05; **, P < 0.01; ***, P < 0.001.

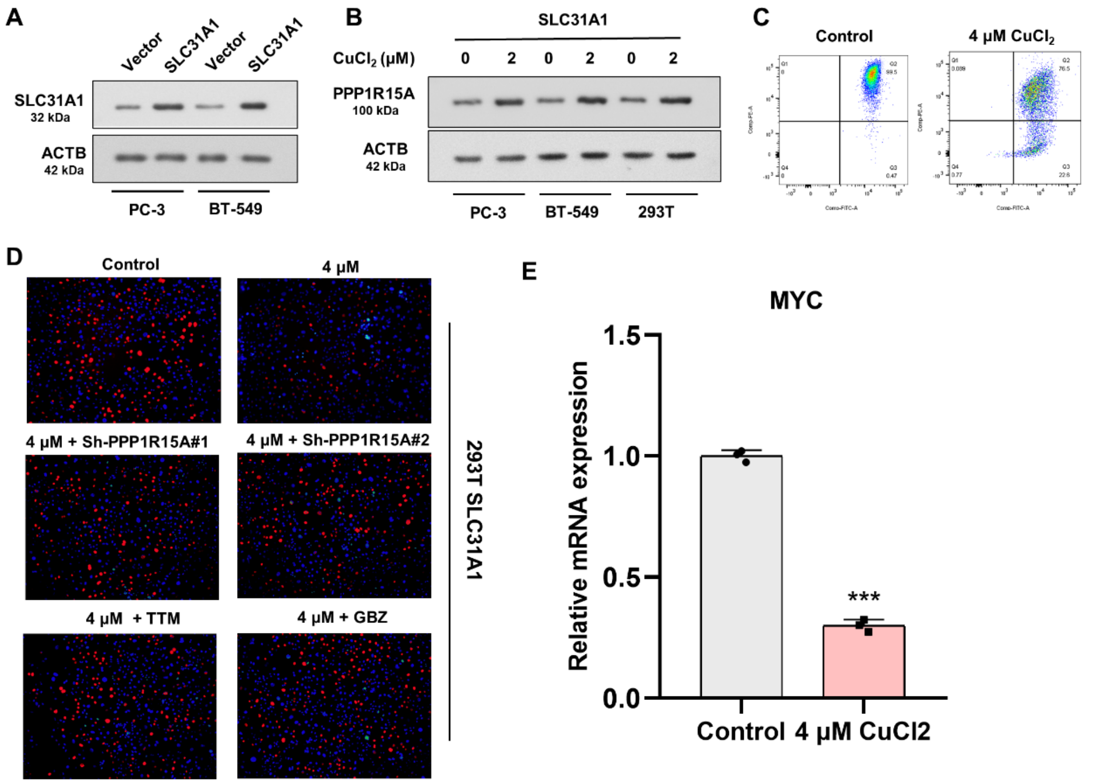

Supplementary Figure 6. Cuproptosis mechanisms are shared in the model of copper homeostasis dysregulation

(A) Protein level of SLC31A1 was assessed by western blot.

(B) Protein content was analyzed and SLC31A1 overexpression cells 24 hours after treatment of supplementation with indicated concentrations of $CuCl_2$.

(C) Flow cytometry assay revealed elesclomol decreased mitochondrial membrane potential in 293T cells stably transfected with SLC31A1.

(D) Representative images of Fig. 7F.

(E) Relative mRNA expression level of MYC under indicated treatment in 293T cells stably transfected with SLC31A1.

Data are presented as the means ± SD from independent experiments. *, $P < 0.05$; **, $P < 0.01$; ***, $P < 0.001$.
