# Supplementary material for: Protein phosphatase 1 regulatory subunit 15 A promotes translation initiation and induces G2M phase arrest during cuproptosis in cancers
Source: Cell Death Dis. 2024 Feb 16;15(2):149. doi: 10.1038/s41419-024-06489-w (PMC10873343; doi:10.1038/s41419-024-06489-w)

Fig. 2A

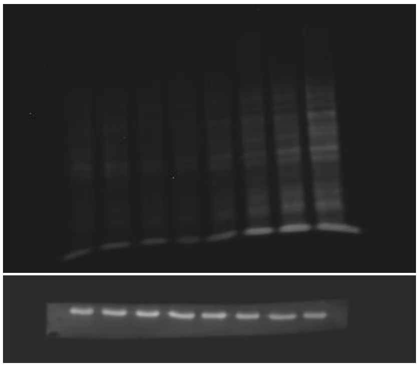

Fig. 2B

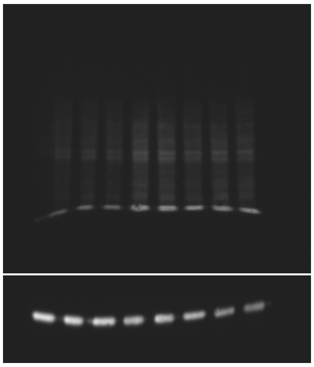

Fig. 2D

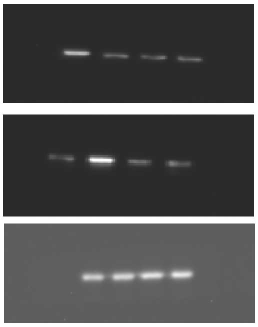

Fig. 2E

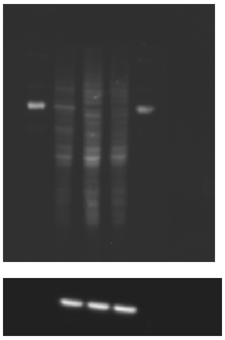

Fig. 2F

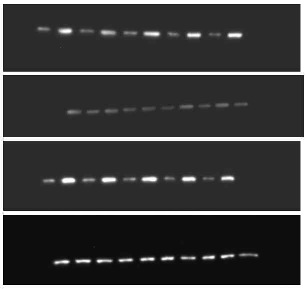

Fig. 2G

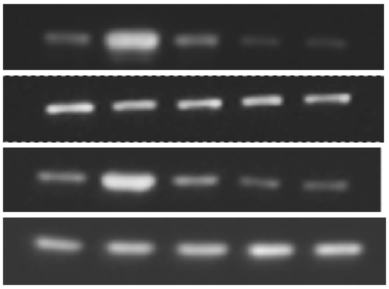

Fig. 3A

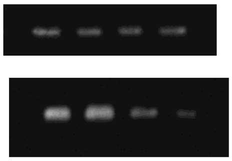

Fig. 3E

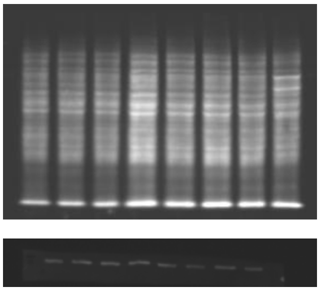

Fig. 3F

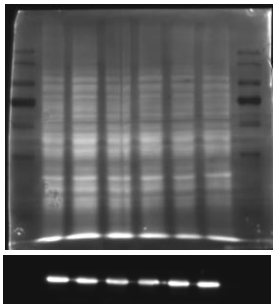

Fig. 4C

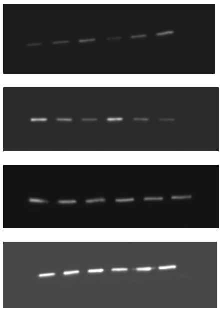

Fig. 4D

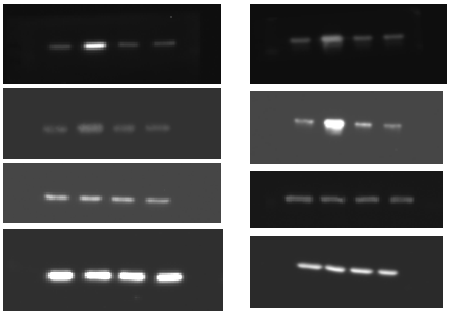

Fig. 4E

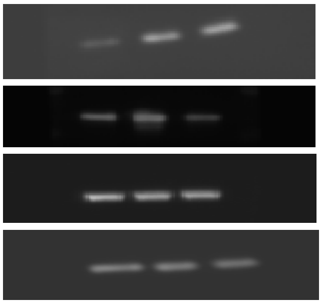

Fig. 4G

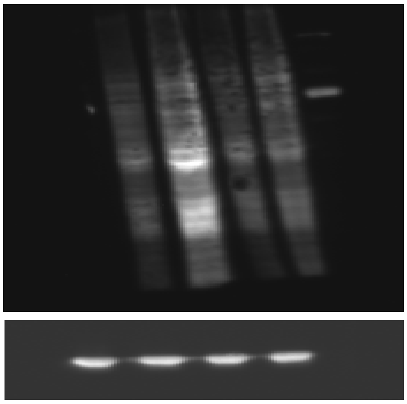

Fig. 4H

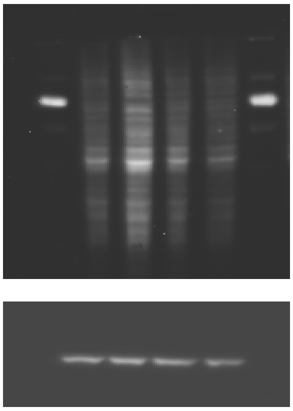

Fig. 6D

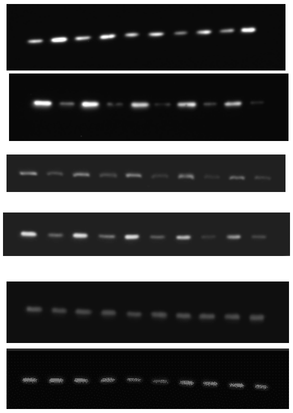

Fig. 6E

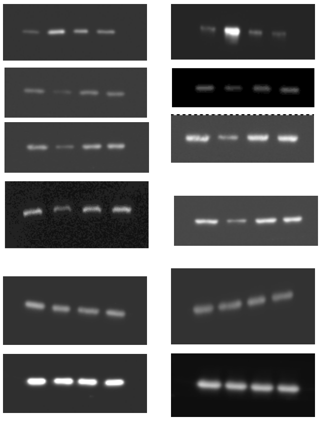

Fig. 6F

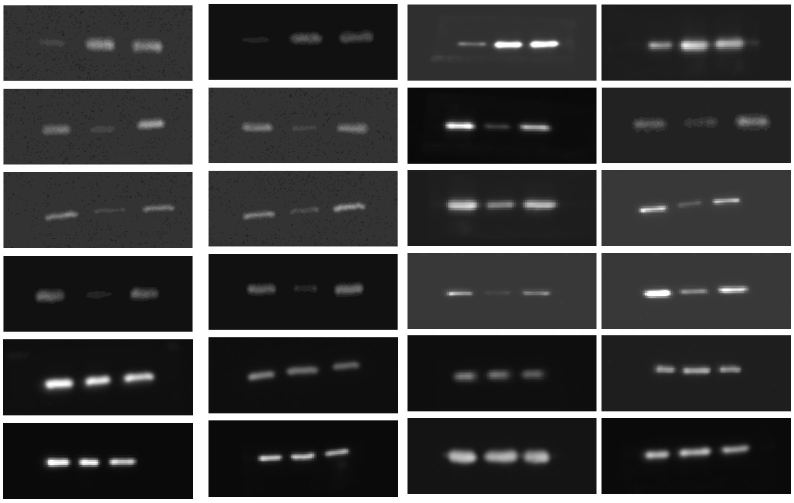

Fig. 7A

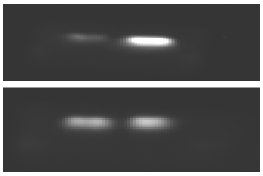

Fig. 7B

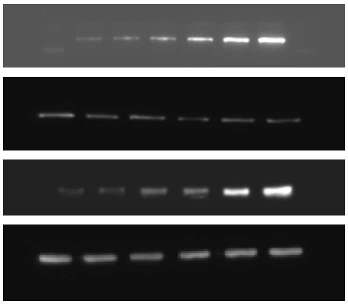

Fig. 7G

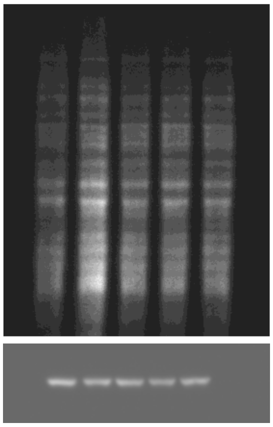

Fig. 7J

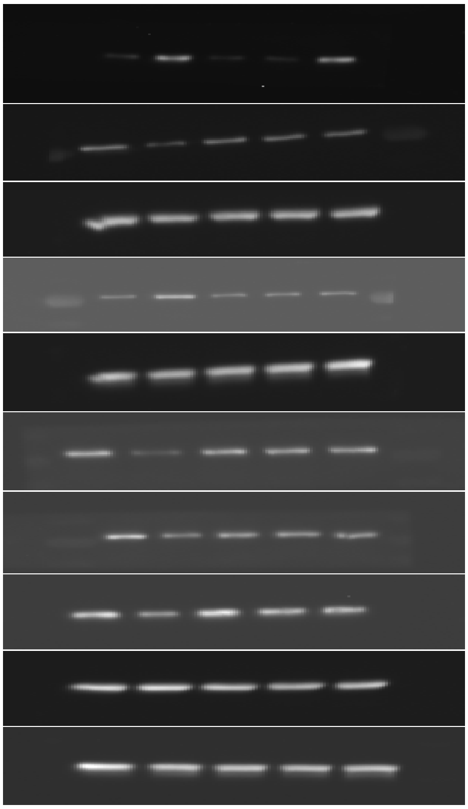

Fig. S2A

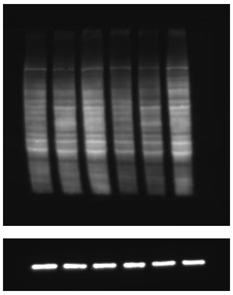

Fig. S2B

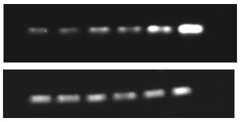

Fig. S3C

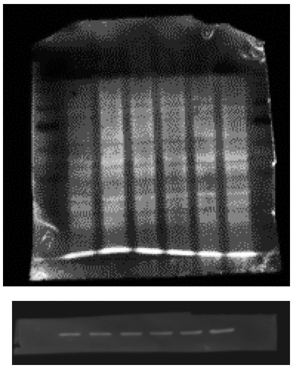

Fig. S2C

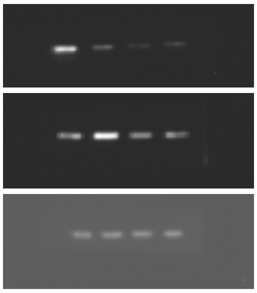

Fig. S3B

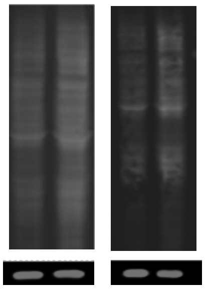

Fig. S4D

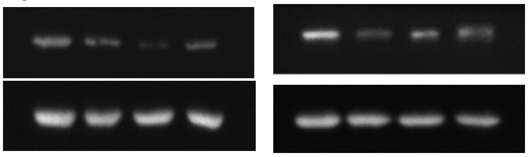

Fig. S2D

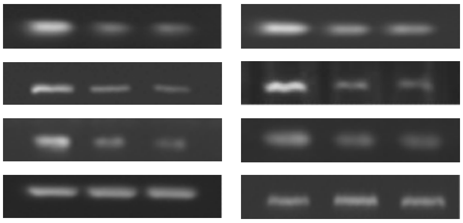

Fig. S2H

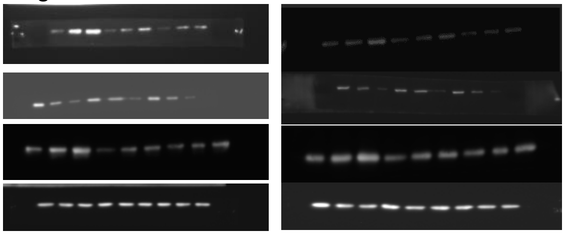

Fig. S5D

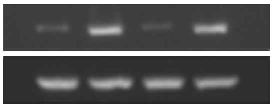

Fig. S4C

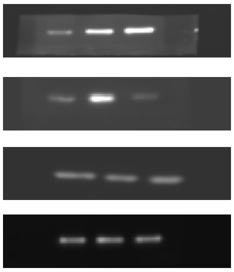

Fig. S6A

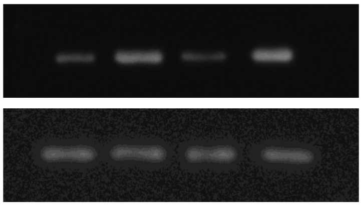

Fig. S6B

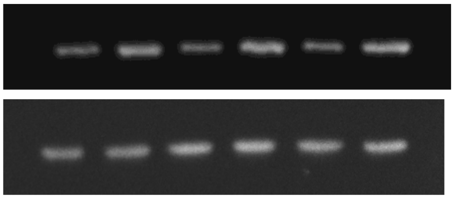

Supplement: Supplementary file 4 — Western Blotting [file 41419_2024_6489_MOESM4_ESM.pdf]
